# Unraveling the Mechanism and Influence of Auxiliary Ligands on the Isomerization of Neutral [P,O]-Chelated Nickel Complexes for Olefin Polymerization

**DOI:** 10.1021/acs.joc.4c02856

**Published:** 2025-01-28

**Authors:** Jeremy Tan, Jingyi Liu, Xinglong Zhang

**Affiliations:** § Department of Chemistry, The Chinese University of Hong Kong, Shatin, New Territories, Hong Kong, China; † Institute of High Performance Computing, Agency for Science, Technology and Research (A^*^STAR), 1 Fusionopolis Way, #16-16 Connexis, Singapore 138632, Republic of Singapore; ‡ Department of Chemistry, National University of Singapore, 4 Science Drive 2, Singapore, 117544, Republic of Singapore

## Abstract

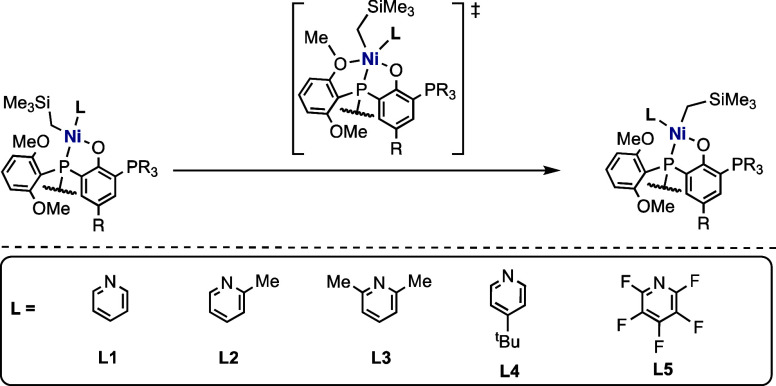

The copolymerization of ethylene with polar monomers presents a significant challenge. While palladium catalysts have shown promise, nickel catalysts are more economical but suffer from poor activity. Previous studies suggest that the isomerization step involved in the nickel-catalyzed polymerization may influence the catalyst activities. Herein, we explore the isomerization mechanisms of two phosphine-phenoxide-ligated catalysts using density functional theory (DFT) studies. We found that out of dissociative, tetrahedral, and associative mechanisms, the associative mechanism is the likeliest, with a pendant methoxy oxygen atom from the ligand to fulfill the fifth coordination site on nickel before Berry pseudorotation. The effect of varying auxiliary ligands on the activation barrier heights was also investigated and found that electron-releasing alkyl groups on substituted pyridine ligands have diminished electronic influence on pseudorotational barriers, but if present at the *ortho*-positions, will elevate the barriers due to larger steric influences. The electron-withdrawing groups on the ligand result in weaker ligand binding and lower pseudorotational barriers. These insights into the mechanisms of *cis-trans* isomerization and auxiliary ligand effects may offer valuable guidance for optimizing catalyst performance in copolymerization processes by lowering the barrier of isomerization by fine-tuning the steric and electronic influences of auxiliary ligands and enhancing overall copolymerization efficiency.

## Introduction

The invention of polymers has greatly revolutionized our life, as polymers find a wide range of applications in modern life. Of the different types of polymers, the incorporation of polar groups can enhance desirable polymer properties such as solubility, adhesion, wettability, and biocompatibility. Polyethylene (PE) is a widely used plastic in a range of industries and accounted for 34% of the entire plastic market in 2017.^1^ Industrial polymerization of ethylene with nonpolar monomers using transition metal catalysts is well-known.^2^ However, the copolymerization of ethylene with polar monomers on an industrial scale is much underdeveloped.^2−4^ This longstanding challenge results from the issue known as the “polar monomer problem”,^3,5^ which occurs when the Lewis basic polar group coordinates to the transition metal center, forming stable chelates and thus poisoning and rendering the catalyst ineffective. As such, there is a dire need for breakthroughs in catalyst development for the copolymerization of polar monomers with ethylene.

**Scheme 1:**
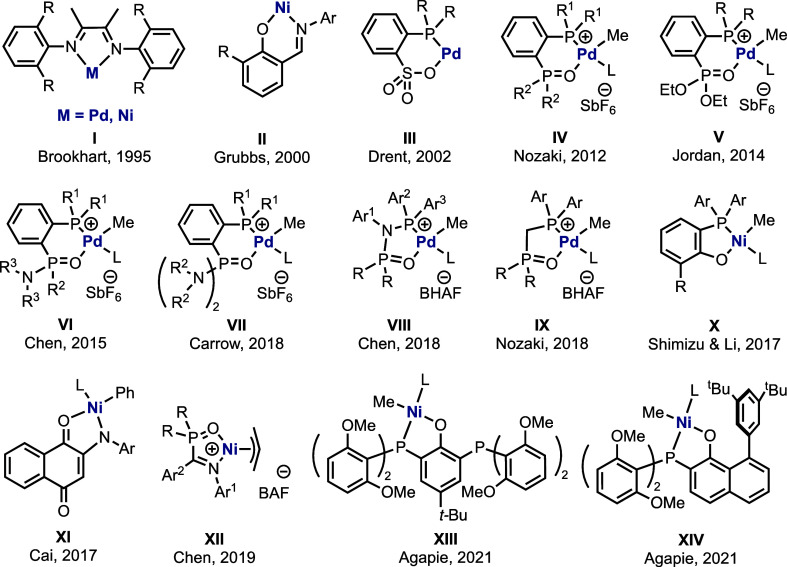
Palladium and Nickel-Based Catalysts for the Copolymerization of Ethylene with Polar Monomers

The pioneering work in the field of polar monomer copolymerization came when Brookhart and coworkers in 1995 reported the use of α-diimine palladium and nickel catalysts (Brookhart catalysts, **I**, Scheme 1) to incorporate polar methyl acrylate and fluorinated octyl acrylate into polyethylenes.^6^ Further advancement in the field came in 2000, when Grubbs and coworkers reported the use of neutral salicylaldimine nickel catalysts (**II**, Scheme 1) for the insertion of polar-substituted norbornenes into polyethylene.^7^ Based on this work, additional reports capitalizing on this type of nickel catalyst for the insertion of other specialized polar monomers into ethylene have appeared in the literature.^8−11^ However, these neutral nickel catalysts have not been applied successfully to copolymerize fundamental polar monomers (where functional group substitution occurs at the C=C bond directly).^4^ Shortly after the report of Grubbs, Drent and coworkers in 2002 showed that palladium catalysts with phosphine-sulfonate bidentate ligands (Drent catalyst, **III**, Scheme 1) can efficiently facilitate ethylene-alkyl acrylate copolymerization.^12^ Based on the Drent systems, a series of cationic Pd catalysts have been proposed (**IV**–**IX**, Scheme 1)^13−18^ and shown to be able to catalyze the copolymerization of either specialized or fundamental polar monomers with ethylene. Of these, the Pd catalysts, with suitable ligand tuning, from Chen and coworkers in 2015 can catalyze ethylene and polar monomer (methyl acrylate, butyl vinyl ether, and allyl acetate) copolymerization with moderate activities (0.5–14 kg·mol^–1^·h^–1^) at tunable comonomer incorporation (0.4–33 mol %), forming moderate copolymer molecular weights (*M*_n_: 2,300–32,000). The corresponding nickel catalysts, however, can only copolymerize ethylene with polar functionalized monomers such as methyl 10-undecenoate, 6-chloro-1-hexene, and 5-acetoxy-1-pentene.^19^

While the palladium-catalyzed copolymerization of olefins with polar monomers has made significant progress, as discussed previously, researchers have increasingly focused on the corresponding transformations using nickel catalysts, which are cheaper and more abundant in nature than palladium catalysts. In 2017, Shimizu and coworkers reported the success of nickel complexes bearing *o*-bis(aryl)-phosphinophenolate ligands (**X**, Scheme 1) in achieving efficient ethylene copolymerization with fundamental polar monomers, including various acrylates.^20^ Again in 2017, Cai and coworkers reported the use of nickel catalysts bearing anilinonaphthoquinone ligands (**XI**, Scheme 1) for the copolymerization of ethylene and specialized polar monomers such as allyl acetate.^21^ In 2019, Chen and coworkers reported a series of imine/phosphine-oxide derived nickel catalysts (**XII**).^22^ However, these catalysts can only copolymerize ethylene with specialized polar monomers such as 6-chloro-1-hexene but not with fundamental polar monomers, whose use either quenched the polymerization or led to polyethylene with no comonomer insertion.

**Scheme 2:**
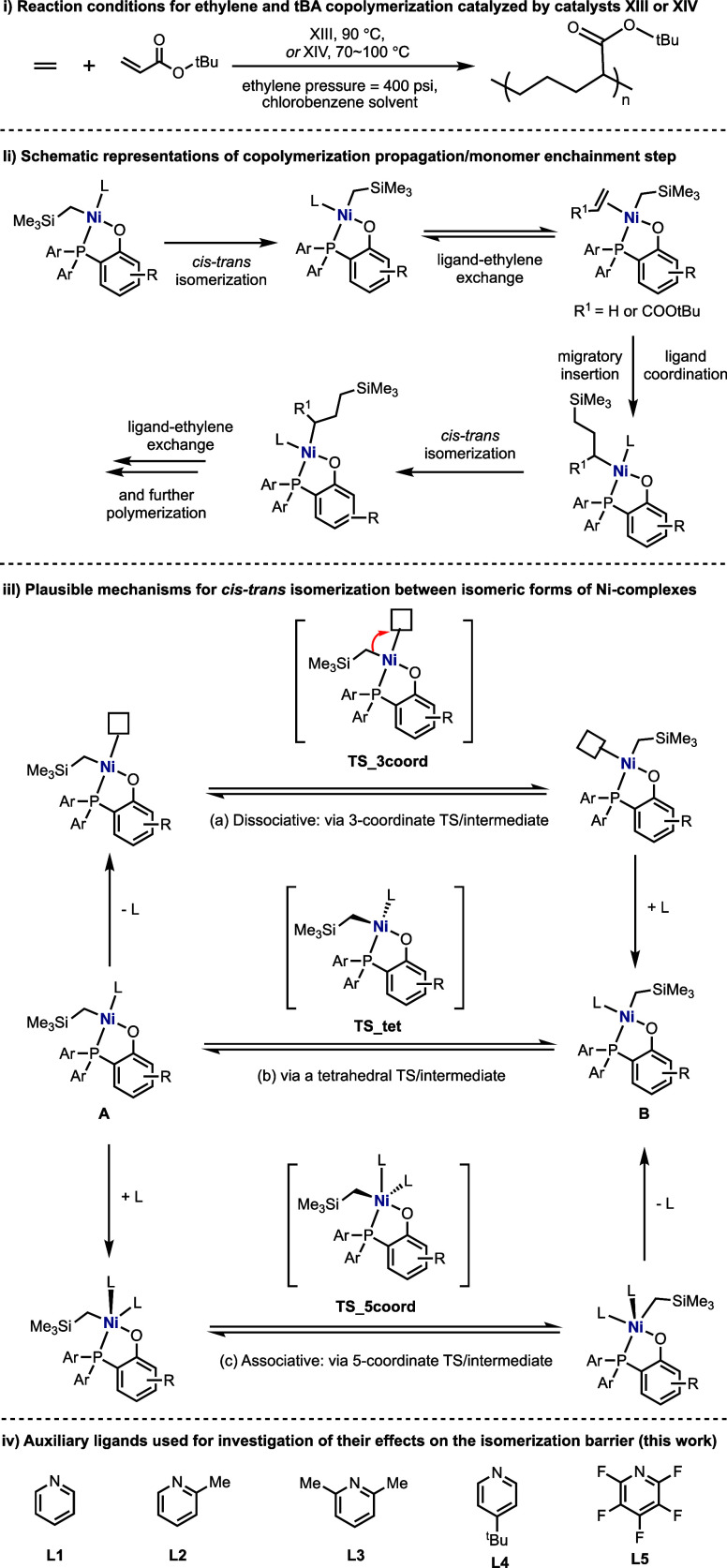
(i) Reaction Conditions for the Copolymerization of Ethylene and Acrylate Using Catalysts **XIII** and **XIV**. (ii) Schematic Representations of the Propagation/Monomer Enchainment Step of Olefin Comonomer Copolymerization. (iii) Plausible Mechanisms for the *cis*-*ttrans* Isomerization Between the Geometric Isomeric Forms of Ni Complexes: (a) Dissociative Mechanism; (b) Via a Tetrahedral Transition State (TS) or Intermediate; (c) Associative Mechanism. (iv) Auxiliary Ligands (**L1** to **L5**) Used for the Investigation of Their Effects on the Isomerization Barriers for the Agapie Catalysts **XIII** and **XIV** in this Study

One of the shortcomings of Ni-based catalysts for polymerization is that they generally suffer from poor activity.^7,20,23−25^ To overcome this issue, the SHOP (Shell Higher Olefin Process)-type [P,O]-Ni catalysts incorporate bulky, electron-rich phosphine substituents to achieve high polymerization activity. These, however, still suffer from poor polar monomer incorporation.^20,26^ In 2021, Agapie and coworkers discovered that by tuning the steric bulk of the [P,O]-ligand from the “O”-side, instead of from the “P”-side as in the SHOP-type Ni catalysts, the modified ligand can function smoothly to prevent bulky polar groups’ inhibitory coordination, while still allowing for the coordination of the smaller olefin group for polymerization to occur.^27,28^ Satisfying these steric demands, two such Ni-based catalysts have been proposed (**XIII** and **XIV**, Scheme 1) for the copolymerization of ethylene and a fundamental polar acrylate comonomer (Scheme 2ii). Catalyst **XIII** (POP-Ni-py) can achieve an activity of up to 661 kg·mol^–1^·h^–1^ and polar comonomer (acrylate) insertion of 2.1–8.7 mol%, forming high copolymer molecular weights of *M*_n_ 31,100–55,100. On the other hand, catalyst **XIV** (PONap-Ni-py) can achieve an activity of up to 637 kg·mol^–1^·h^–1^ and polar comonomer (acrylate) insertion of 0.7–2.3 mol%, forming moderate copolymer molecular weights of *M*_n_ 7,600–16,500.

Of the key steps for the ethylene and acrylate copolymerization, it was found that a crucial *cis-trans* isomerization occurs at initiation and each step after a monomer has been incorporated (Scheme 2ii). Due to the asymmetric nature of the Ni-ligand complexes **XIII** and **XIV**, two geometric isomers with elongating chains either *cis* or *trans* to the P atom can be formed. Computational studies have found that the more stable isomer has a higher barrier for C–C bond formation via migratory insertion in the chain elongation process, whereas the less stable isomer has a lower barrier, making the process of isomerization from the more stable to the less stable species a prerequisite before migratory insertion.^27^ This phenomenon has also been observed in Pd-catalyzed polymerization processes utilizing different ligands.^29−33^ The mechanism for this *cis-trans* isomerization is thus important for understanding the performance of the catalysts. The importance of main ligands and the auxiliary ligands (ligand **L** in Schemes 1 and 2) in tuning the efficiency and reactivities of the polyolefin catalysts has been well demonstrated, wherein ligand tunings are often needed to optimize catalyst performance.^13−18,20,22^ In this article, we apply computational tools to study the mechanisms underlying the *cis-trans* isomerization (Scheme 2iii) for the Agapie catalysts (**XIII** and **XIV**) and determine how the auxiliary ligands influence the activation barriers for the isomerization process (Scheme 2iv). Understanding the detailed mechanism for this step allows experimentalists to fine-tune the catalyst designs so as to minimize the energetic penalties involved for this step, allowing the main C–C coupling to occur more efficiently, thus enhancing the catalyst activity and performance.

## Results and Discussion

### Gibbs Energy Profile for Monomer Insertion

**Figure 1:**
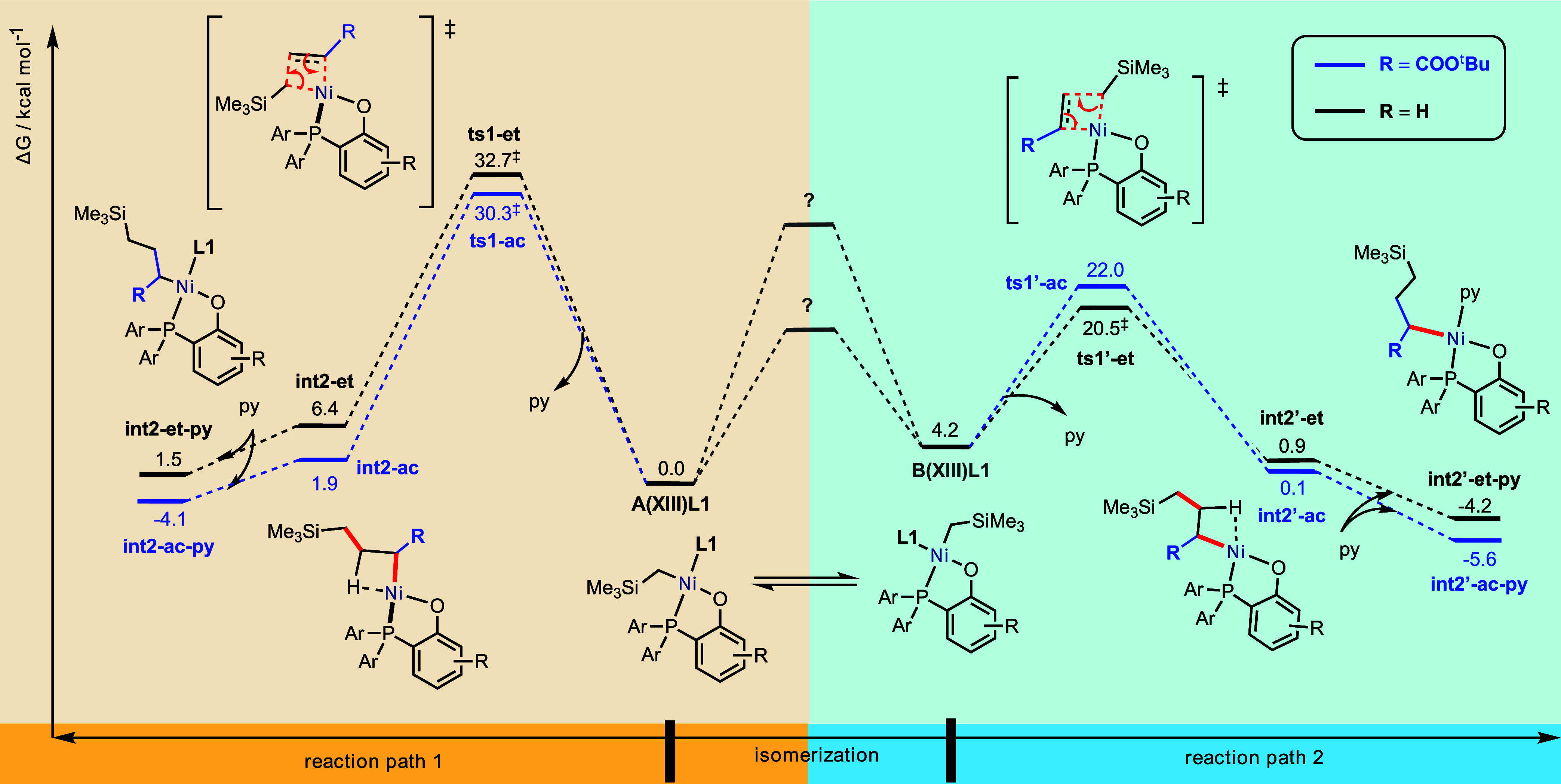
Gibbs energy profile for the first monomer insertion steps leading from each isomeric form **A(XIII)L1** and **B(XIII)L1**, calculated at the SMD(chlorobenzene)-M06/def2-TZVP//SMD(chlorobenzene)-M06/def2-SVP level of theory. See Figure S1 for the DFT-optimized structures of the transition states. Note the importance of the isomerization step, as the barrier determines if this step is higher or lower than the polymerization step.

The Gibbs energy profile for the first monomer insertion step catalyzed by catalyst **XIII** for the reaction in Scheme 2ii is shown in Figure 1. For this work, all geometry optimizations were done in SMD^34^ implicit solvation model with chlorobenzene as the solvent with M06^35^/def2-SVP^36,37^ level of theory. Single-point energy corrections were further performed with the SMD(chlorobenzene)-M06/def2-TZVP^37^ level of theory. Unless otherwise stated, the Gibbs energies reported were evaluated at the SMD(chlorobenzene)-M06/def2-TZVP//SMD(chlorobenzene)-M06/def2-SVP level of theory (see the Supporting Information for details on the computational methods). Despite the complex **A(XIII)L1** being more thermodynamically stable than species **B(XIII)L1** by 4.2 kcal/mol, the monomer insertion transition states (TSs) from complex **A(XIII)L1**, **ts1-et** for ethylene insertion and **ts1-ac** for acrylate insertion, have much higher barriers than the corresponding monomer insertion from the less stable complex **B(XIII)L1**, via TSs **ts1′-et** and **ts1′-ac**, by 12.2 and 8.3 kcal/mol, respectively (Figure 1). It is therefore important to study the reaction mechanism for the isomerization step between complexes **A(XIII)L1** and **B(XIII)L1**, as this step is essential to convert the thermodynamically more stable but less reactive species **A(XIII)L1** to the less stable but more reactive species **B(XIII)L1** before the monomer insertion step occurs. We have shown in our previous work^27^ that this is similarly observed for the second monomer insertion step. For these Agapie catalysts, the growing polymer chain *cis* to the P atom (structure **A**, Scheme 2iii) is more thermodynamically stable than the geometric isomer with the chain *trans* to the P atom (structure **B**, Scheme 2iii). Structure **A**, however, subsequently has a much higher C–C bond formation barrier than structure **B** for the polymer elongation step.^27,33^ Therefore, structure **A** will isomerize to form **B** before further polymer enchainment. Note that this isomerization will be repeated each time after C–C bond formation (Scheme 2ii). Thus, the barrier of the isomerization step affects the overall polymerization of the catalyst performance. In Figure 1, if the isomerization step is higher than polymerization, then it is important to lower this step so that it does not become overall rate limiting. Therefore, by studying the mechanism of isomerization and aiming to lower the activation barrier for this step using suitable coligands, we aim to increase the efficiency of the catalyst performance in the polymerization process.

### Mechanism of *cis-trans* Isomerization

#### Catalyst XIII

As shown in Scheme 2iii, three mechanistic possibilities exist for the *cis-trans* isomerization between the geometric isomers of the Ni complexes with a growing polymer chain. In the dissociative mechanism (Scheme 2iii,a), the auxiliary ligand **L** dissociates, giving a vacant coordination site on the Ni center. The growing polymer chain then moves from its original coordinating site to its adjacent newly vacated coordination site. This is then followed by the coordination of the ligand **L** to the site previously occupied by the growing polymer chain. In the second mechanism (Scheme 2iii,b), the square planar structure **A** will twist and go through an isomerization transition state (TS) that is tetrahedral in nature. Lastly, in an associative mechanism (Scheme 2iii,c), a fifth ligand may coordinate to the Ni center, giving a penta-coordinate species. This can then undergo a Berry pseudorotation to isomerize the catalyst from one geometric isomer to the other, before finally releasing one ligand to revert to the square planar geometric isomer species.

**Figure 2:**
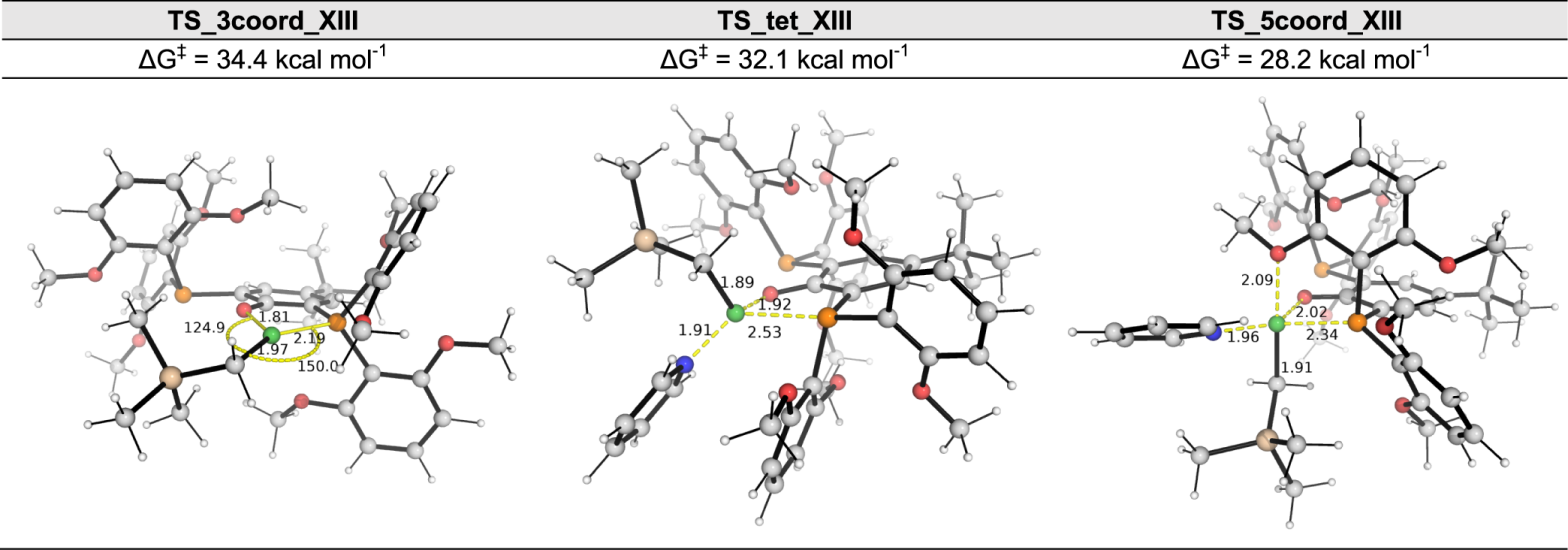
DFT-optimized transition state (TS) structures for the different mechanisms for the isomerization of **A(XIII)L1** to **B(XIII)L1**: dissociative, **TS_3coord_XIII**; tetrahedral, **TS_tet_XIII**; associative, **TS_5coord_XIII**. All Gibbs energies are taken relative to the most stable form of **A(XIII)L1**. See Figure S3 for other TS conformers.

In the first investigation of the isomerization process, Agapie catalyst **XIII** and pyridine ligand were used (**L**=**L1**=pyridine, Scheme 2iv). We denote this structure as **A(XIII)L1**, where structure **A** in Scheme 2iii with catalyst **XIII** was used (equivalently, catalyst **XIII** in Scheme 1 has the Me group replaced by the −CH_2_SiMe_3_ group). We first tried to estimate the barrier to pyridine dissociation by doing a relaxed PES scan along the elongated Ni–N(pyridine) bond. The SMD(chlorobenzene)-M06/def2-SVP relaxed potential energy surface (PES) scan is given in Figure S2. We see that the loss of the pyridine ligand is unfavorable. As the Ni–N(pyridine) bond length increases, the energy of the system increases. This increase continues until about 27 kcal/mol when the Ni–N(pyridine) bond is 3.36 Å, after which the energy drops. This scan suggests that the barrier for pyridine ligand dissociation is about 27 kcal/mol. Upon further increasing the Ni–N(pyridine) bond distance, the energy increases again, suggesting that the removal of the ligand is unfavorable, as the Ni–N(pyridine) enthalpic interaction is lost upon ligand dissociation. This loss of pyridine is uphill and reversible, corroborated by the observation that starting from the initial guess structure of a long Ni–N(pyridine) bond (arbitrarily large at 3.70 Å by manually increasing this distance in the corresponding structure **A(XIII)L1**, while maintaining the square plane of the Ni center), this optimized back to structure **A(XIII)L1**. The optimization of structure 4 from the PES scan (Figure S2) gives optimized structure **A1a**, in which the Ni–N(pyridine) interaction is lost (Figure S3). This species is 22.4 kcal/mol above **A(XIII)L1**, indicating that the loss of Ni–N(pyridine) coordination is highly unfavorable. Using the structure at the highest point of this PES scan as the initial guess to locate the TS for ligand dissociation yielded no positive results. The TS structure for the isomerization of the three-coordinate system after losing ligand **L** (**TS_3coord**, Scheme 2iii), however, was successfully located. This TS has a barrier of 34.4 kcal/mol (Figure 2). Note that this result is very close (within 1 kcal/mol) to the reported value for this system (33.8 kcal/mol) computed at the SMD(chlorobenzene)-M06/def2-TZVP//M06/def2-SVP level of theory using geometry optimizations in the gas phase.^27^

In the associative mechanism (Scheme 2iii,c), we first consider if an external ethylene molecule could serve as the fifth ligand by binding to the Ni center. The direct optimization using ethylene coordinated to the Ni center as an initial guess structure did not yield a stable 5-coordinate species. This species **A-et**, with ethylene unbound (Figure S3), is 6.5 kcal/mol uphill with respect to the most stable form of complex **A(XIII)L1**. This is due to the unfavorable entropic effect associated with bringing in an additional ethylene molecule to the inner coordination shell of the Ni metal, while this loss of entropy cannot be compensated by any enthalpic favorability, as no Ni–ethylene interaction is formed. The absence of a stable penta-coordinate Ni species with bound ethylene suggests that the coordination of ethylene to form 5-coordinate species is unlikely. A similar observation was made computationally when a chlorobenzene solvent molecule was used as the candidate coordinating ligand. We found that in the TS structure, the O atom of the methoxy group on the ligand can serve as the fifth ligand in coordinating the Ni center. Two TS conformers for the pseudorotational barriers (**TS_5coord_XIII** in Figure 2 and **TS_5coord_XIII-c2** in Figure S3) were found and verified by quick reaction coordinate (QRC)^38^ to be the true TS structures for the isomerization of one catalyst form (**A(XIII)L1**) to its geometric isomer (**B(XIII)L1**). The lowest energy conformer **TS_5coord_XIII** has a relatively shorter Ni–OMe bond distance (2.09 Å, Wiberg bond index 0.2054, Figure 2) that is comparable to the Ni–O(Ph) bond distance (2.02 Å, Wiberg bond index 0.3373), suggesting that the coordination of the methoxy oxygen atom to the Ni center acts as a fifth ligand. This TS has a barrier of 28.2 kcal/mol, which is lower than both the barriers via the three-coordinate TS (**TS_3coord_XIII** at 34.4 kcal/mol, Figure 2) and the tetrahedral TS (**TS_tet_XIII** at 32.1 kcal/mol, Figure 2). This penta-coordinate TS with an intramolecular pendant oxygen atom as the fifth coordinating ligand benefits both entropically, as no external molecule is brought into the coordination sphere of Ni, and enthalpically, as a favorable Ni–O bonding interaction is formed.

We further analyzed the frontier molecular orbitals (FMOs) of the competing TSs (Figure S4). We hypothesize that the higher barrier for **TS_3coord_XIII** may be due to the breaking of d-σ orbital interactions as one chain sweeps from one coordination site to the other. For **TS_tet_XIII**, there is a twist of geometry in order to go from one isomer to the other, resulting in the change of orbital phases in the Ni(d)–C(σ) orbital and Ni(d)–N(lone pair) orbital interactions, which is unfavorable. For the orbitals involved in the pseudorotational barrier via **TS_5coord_XIII**, the orbital interactions are very much preserved as isomerization occurs, thus giving it a much lower activation barrier.

**Figure 3:**
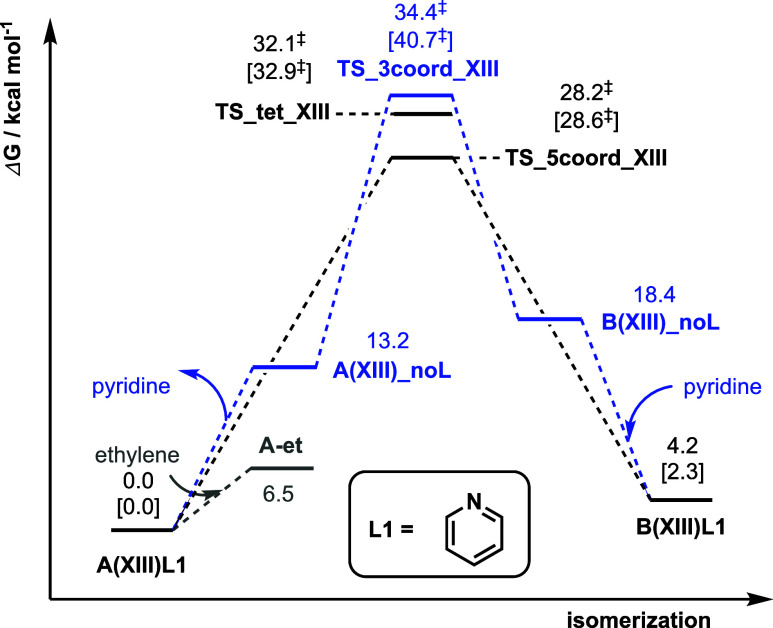
Gibbs energy profile for the isomerization of **A(XIII)L1** to form **B(XIII)L1**. Values in square brackets are computed at the SMD(chloroform)-DLPNO–CCSD(T)/CBS//SMD(chloroform)-M06/def2-SVP level of theory to verify the DFT methods.

This associative mechanism is therefore the likeliest: the isomerization of catalyst complex **A(XIII)L1** to its isomeric form **B(XIII)L1** occurs via an associative mechanism with a *proximal OMe group serving as a fifth coordinating ligand* to the Ni center before a Berry pseudorotation isomerizes the complexes. The other possibility of losing P-coordination to O-coordination by the methoxy group (thus losing the P,O-chelating framework) was considered and found to be unlikely (structure **A1c** at 30.2 kcal/mol above **A(XIII)L1**, Figure S3). This is perhaps expected as the loss of stronger Ni–P coordination was replaced by the weaker Ni–O (methoxy) interaction, and the 8-membered Ni-ring bound by the POP ligand in a bidentate fashion via phenoxide O and methoxy O atoms suffers from unfavorable ring strains. Figure 3 summarizes the Gibbs energy barriers for all of the mechanistic possibilities for the isomerization step involving catalyst **XIII**.

#### Catalyst XIV

**Figure 4:**
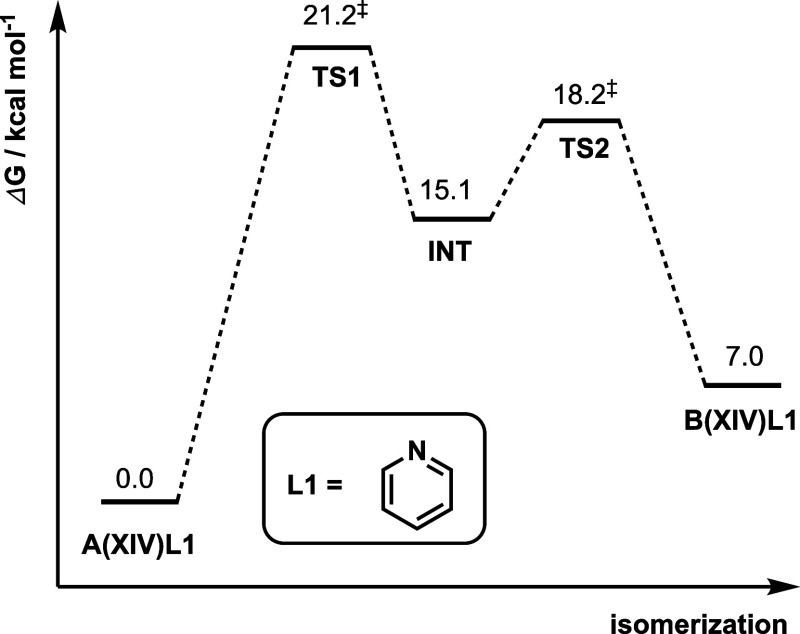
Gibbs energy profile for the isomerization of **A(XIV)L1** to **B(XIV)L1** via a two-step process. **TS1** is renamed to **TS_5coord_XIV** as it is the highest barrier step on which we focus on.

We next investigated the isomerization mechanism for Agapie catalyst **XIV**. We first considered the complex when the pyridine ligand was used (**L**=**L1**=pyridine, Scheme 2iv). We considered the isomerization of complex **A(XIV)L1**, formed from catalyst **XIV** in Scheme 1 by replacing the Me group with the −CH_2_SiMe_3_ group, to its geometric isomeric complex **B(XIV)L1**. For this isomerization process, interestingly, the penta-coordinate transition step undergoes a two-step pseudorotation through two distinct transition states. The Gibbs energy profile is shown in Figure 4 with the associated DFT-optimized structures shown in Figure S5. The first step involves the coordination of the pendant methoxy oxygen, as previously described, that passes through **TS1** that has a trigonal bipyramidal shape. This TS primarily moves the alkyl side chain from the equatorial position to the axial position, giving a stable intermediate **INT** (DFT-optimized structure in Figure S5). In the second step, via **TS2** (Figure S5), the pyridine ligand swings over the alkyl group so that it goes from its original equatorial position *trans* to the P atom to the adjacent equatorial position *cis* to P, thereby giving the isomerized structure. We note, however, that the first step has a higher barrier than the second step, by 3.0 kcal/mol. Therefore, we focus on the first step as the rate-determining step for isomerization via the penta-coordinate mechanism. We rename **TS1** as **TS_5coord_XIV** for further discussion.

**Figure 5:**
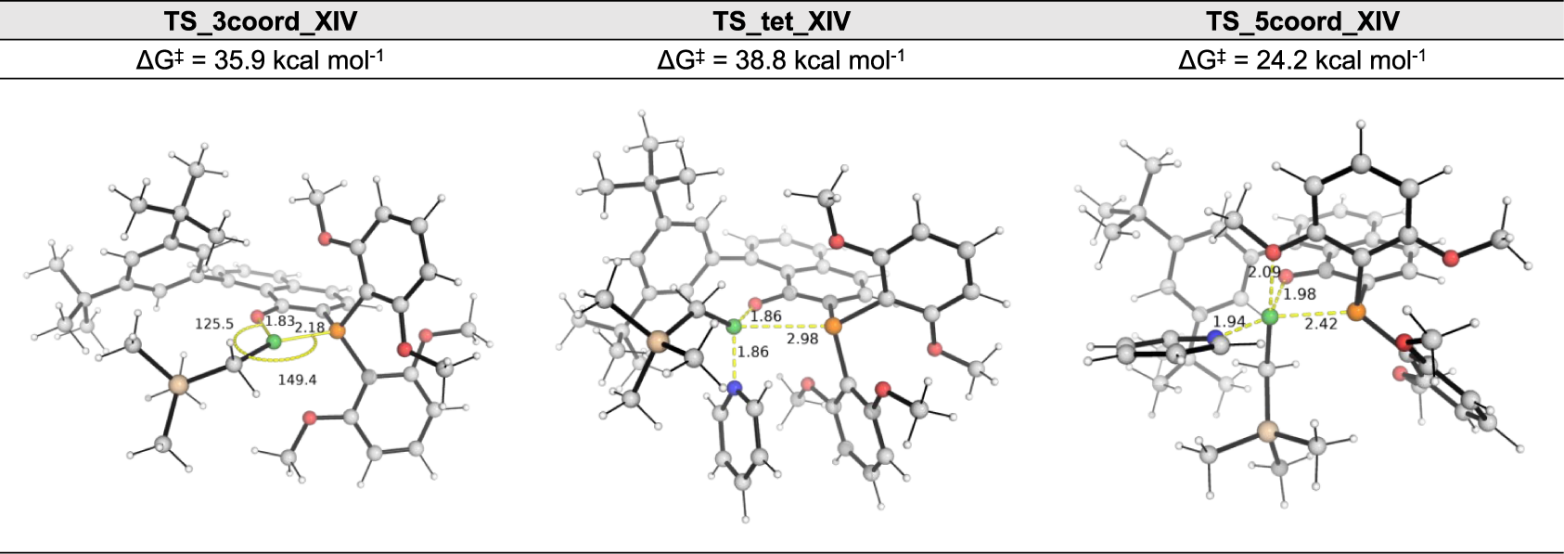
DFT-optimized transition state (TS) structures for the different mechanisms for the isomerization of **A(XIV)L1** to **B(XIV)L1**: dissociative, **TS_3coord_XIV**; tetrahedral, **TS_tet_XIV**; associative, **TS_5coord_XIV**. All Gibbs energies are taken relative to the most stable form of **A(XIV)L1**.

Other mechanistic possibilities via a dissociative, 3-coordinate transition state and via a tetrahedral transition state were also considered. The key TS structures for the mechanistic possibilities are shown in Figure 5. We observe that for this system, similar to the **XIII** system, the intramolecular associative mechanism (via **TS_5coord_XIV**, 24.2 kcal/mol, Figure 5) wherein the coordination of a pendant methoxy O atom forming a penta-coordinate TS that involves Berry pseudorotation is favored, as seen in **the XIII** system, over both the dissociative mechanism (via **TS_3coord_XIV**, 35.9 kcal/mol, Figure 5) and the tetrahedral mechanism (via **TS_tet_XIV**, 38.8 kcal/mol, Figure 5).

#### Comparison of Penta-Coordinate TSs for Catalysts XIII and XIV

**Figure 6:**
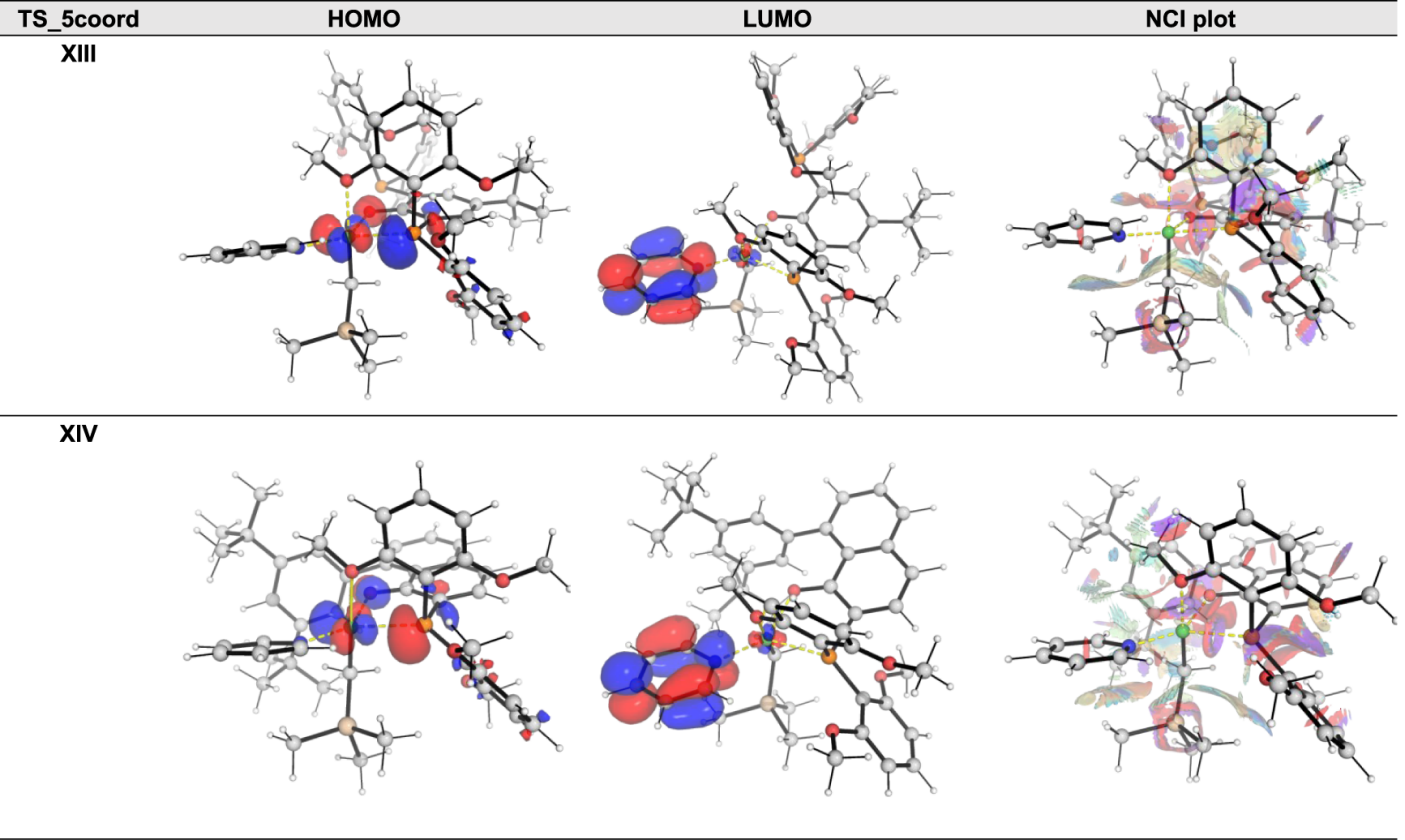
Frontier molecular orbital (FMO) plots including HOMO and LUMO and the noncovalent interaction (NCI) plots for **TS_5coord_XIII** and **TS_5coord_XIV**. Molecular orbitals are visualized using an isosurface value of 0.05 au NCI indices calculated with NCIPLOT^39^ were visualized at a gradient isosurface value of *s* = 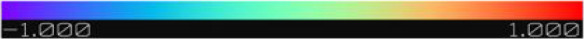 0.5 au. The color range is such that blue is attractive (−1.000) and red is repulsive (+1.000).

We endeavored to understand the differences in the catalyst systems **XIII** and **XIV** by focusing on the penta-coordinate transition states, **TS_5coord_XIII** and **TS_5coord_XIV**. We note that the latter has a barrier that is 4.0 kcal/mol lower than that of the former. Frontier molecular orbitals (FMOs) analysis suggests that the orbital coefficients in the HOMO and LUMO in both TSs are similar (Figure 6). Natural bond orbital (NBO) charge analysis indicates that the charges at the Ni center and O centers are similar for both TSs: Ni is positively charged at +0.528 in **TS_5coord_XIII** and at +0.559 in **TS_5coord_XIV** (Table S3); similarly, the NBO charge of the phenoxide oxygen atom is −0.726 in **TS_5coord_XIII** and −0.709 in **TS_5coord_XIV** (Table S3). Although similar frontier MO structures and NBO charges are observed, these indicate similar orbital and electronic interactions in these TSs. A higher electron donation from the phenoxide oxygen to the Ni center, as indicated by the more negative/larger second-order perturbative stabilization energy, E(2), is observed in **TS_5coord_XIV** (138.9 kcal/mol) than in **TS_5coord_XIII** (128.0 kcal/mol) (Table S4). This additional stabilization of 10.9 kcal/mol may contribute to the lower activation barrier observed in **TS_5coord_XIV**. NCI plots showing noncovalent interactions are also shown in Figure 6. They indicate that there is some steric repulsion in the phosphine-phenoxide ligand in catalyst **XIII** that may also contribute to its higher isomerization barrier.

### Role of Auxiliary Ligands on Intramolecular Associative TS Barriers

We next focus on the intramolecular associative mechanism for both Agapie catalysts **XIII** and **XIV** to understand how the identity of these auxiliary ligands (Scheme 2iv) affects the TS barriers. These ligands have been shown to have differing binding affinities to the Ni center,^28^ thus affecting their ability to dissociate and vacate for the olefin/acrylate monomer to be coordinated for subsequent chain propagation. We computed the logarithm of the ratio of equilibrium constants, log(*K*_L_/*K*_py_) and compared it to the values measured experimentally (Table 1). We note that the computed values are close to, but not exactly the same as, the experimentally measured values. The correlation coefficients for these two systems are reasonable, with 0.68 for catalyst **XIII** and 0.73 for catalyst **XIV** (Figure S6). The difference may be due to the methods used experimentally, where both ligands under study are used to obtain the integration of Ni-CHR resonance in the ^1^H/^31^ P NMR resonances, which differ from computed values obtained from binding energy calculations for individual systems (SI section 6). However, despite the different methods, it is worth noting that both experiments and computations pinpoint ligand **L5**, perfluoropyridine, as the ligand that binds the Ni center the weakest, consistent with the conventional wisdom that the N-lone pair on **L5** will be less donating due to the multiple electron-withdrawing F atoms present. It should be noted that substituents on the pyridine ligand can also introduce unfavorable sterics that hinder ligand binding, especially if the substituents are *ortho* to the N atom, such that they may sterically clash with the phosphine-phenoxide ligand. These could account for the experimental observation that despite having electron-releasing methyl substituents on the *ortho*-position (**L2** and **L3**), weaker binding is observed both experimentally and computationally (Table 1). For substituents at *para*-position, for example, **L4** in catalyst **XIII** measured experimentally, a stronger binding to the Ni center than pyridine is observed (Table 1), as the steric influence/clashes with the phosphine-phenoxide ligand are minimized. We note that some disagreement between the computed and experimental log(*K*_L/py_) values may arise due to experimental errors or incomplete conformational sampling during computational studies.

**Table 1:** Experimentally Measured (exp) and Computed (comp) Ligand Competition Values Using Logarithm of Ratio of Equilibrium Constants, log(*K*_L_/*K*_py_) Where py is the Pyridine Ligand (**L1**) and L is Other Ligands (**L2–L5**) Used in Competition Studies

log(*K*_L/py_)	L1	**L2**	**L3**	**L4**	**L5**
**XIII**^**exp**^	0	–1.8	–3.9	0.2	–4.3
**XIII**^**comp**^	0	–1.1	–2.3	–0.1	–7.5
**XIV**^**exp**^	0	–1.8	–4.4	–0.2	–5.1
**XIV**^**comp**^	0	–0.9	–3.6	–1.6	–8.9

We next study the rotational barriers for isomerization via a penta-coordinate transition state for each catalytic system with different auxiliary ligands present. Table 2 gives the computed activation barriers for each system. We note that, within each catalyst system, we have kept the conformations consistent: the pyridine ligand in the located TSs (**TS_5coord_XIII** and **TS_5coord_XIV**) is modified to other ligands without changing the orientation/conformation of all other atoms. The TSs using auxiliary ligands **L2** and **L4** (with electron-releasing alkyl substituents on the pyridine core) give lower barriers than the TS with an unmodified pyridine ligand (**L1**) for both catalysts **XIII** and **XIV** (Table 2). These lowered barriers correlate to the weaker binding affinity of **L2** and **L4** than the pyridine **L1** ligand (Table 1). No systematic trend was observed when comparing catalysts **XIII** and **XIV**, as **TS_5coord_XIII_L2** is the lowest among the three ligands used in **XIII,** but **TS_5coord_XIV_L4** is the lowest in **XIV**. This difference may be due to the differences in the steric demand of the phosphine-phenoxide ligands in **XIII** and **XIV** (Figure S8). It is interesting to note that, despite multiple trials (e.g., modifying from located true TSs, using combinations of dihedral angle/angle/distance scans, using the nudged elastic band (NEB) method), no successful TSs could be located for catalysts with ligands **L3** and **L5** (only methyl rotations are obtainable). We hypothesize that for **L3**, it is likely that the presence of two methyl groups at the *ortho*-position results in significant steric clashes in the way of pseudorotation such that such a TS could not be located. For **L5**, the weak binding arising from the electronic influences of multiple F atoms may cause the weakly bound **L5** ligand to dissociate and render pseudorotation difficult.

**Table 2:** Computed Gibbs Energy Barrier for the Isomerization of Each Catalyst System with Different Ligands^a^

**Catalyst**	**L1**	**L2**	**L3**	**L4**	**L5**
**XIII**	28.2	26.0	–	27.5	–
**XIV**	24.2	23.5	–	22.7	–

a Values are given in kcal/mol.

We proceeded to estimate the barriers for isomerization via the pentacoordinate transition state using ligands **L3** and **L5** (SI section 6), which suggests that the pseudorotational barrier will be higher for **L3** and lower for **L5** than for the pyridine ligand **L1** (Table S2). The FMO plots for these TSs and approximate TSs are similar for all ligands (except that for **L5**, the LUMO has some orbital coefficients on the F atoms) (Figure S8). The NBO charges on the key atoms are similar (Table S3, Figures S9 and S10). We note the lower second-order perturbative stabilization energy, E(2), resulting from the electron donation from the nitrogen atom on the ligand to the Ni center in catalysts **XIII** and **XIV** when the ligand is **L5** (77.9 and 75.7 kcal/mol, for **XIII** and **XIV**, respectively) than other ligands **L1–L4** (Table S4). In replacing pyridine ligand **L1** with **L5**, the NBO charge on the N atom of the ligand becomes more negative by 0.080 au in catalyst **XIII** and by 0.081 au in catalyst **XIV** (Table S3 and Figures S9 and S10); the E(2) value for N → Ni donor–acceptor interaction decreases by 11.6 kcal/mol in **XIII** and by 12.8 kcal/mol in **XIV** (Table S4, Figures S11 and S12). These observations are consistent with the electron-withdrawing nature of the perfluoropyridine ligand and the weakest binding strength observed experimentally and computationally.

## Conclusion

The mechanisms underlying the isomerization processes of neutral phosphine-phenoxide-ligand-coordinated nickel complexes were elucidated through computational DFT studies. Our findings suggest that an associative mechanism with a pendant oxygen from the methoxy group of the phosphine-phenoxide ligand acting to fulfill the fifth coordination site on nickel is essential to enable the isomerization to occur via penta-coordinate Berry pseudorotation. Either single-step or two-step pseudorotations can occur, depending on the identity of and the steric demands on the phosphine-phenoxide ligands. Our investigations into the effect of varying auxiliary ligands reveal that ligands with electron-releasing alkyl groups have electronic influences that may be counterintuitive: with more electron donation to the nickel center and enhanced bonding interaction, the pseudorotational TS barrier is expected to be increased. However, with alkyl-releasing substituents in **L2** and **L4**, the barriers are actually lowered (Table 2). In addition, substituents at the *ortho*-positions may augment the steric clashes and result in higher barriers. Electron-withdrawing substituents on the pyridine ligand are found to weaken the Ni–N binding strength, thus potentially leading to lower barriers. These findings point the way toward a potential strategy for optimizing catalyst performance in copolymerization processes by lowering the barrier of isomerization by fine-tuning the steric and electronic influences of auxiliary ligands, so that the isomerization step is not rate limiting, allowing polymerization to proceed more efficiently. Additional work to study more diverse substituents on the auxiliary ligands with varying strengths of electron-withdrawing and -donating powers may thus pave the way for the design and engineering of better Ni-based copolymerization catalysts.

## Data Availability

The data underlying this study are available in the published article, in its Supporting Information, and openly available on Zenodo at https://zenodo.org/records/14186325, DOI:10.5281/zenodo.14186325 (DFT-optimized structures and associated data).

## Supporting Information Available

DFT details, computational methods, optimized geometries, and additional figures. The authors also have cited additional references^35−58^ (PDF)

## References

[ref1] Geyer R.; Jambeck J. R.; Law K. L. Production, use, and fate of all plastics ever made. Sci. Adv. 2017, 3 (7), e1700782 10.1126/sciadv.1700782. 28776036 PMC5517107

[ref2] Luckham S. L. J.; Nozaki K. Toward the Copolymerization of Propylene with Polar Comonomers. Acc. Chem. Res. 2021, 54 (2), 344–355. 10.1021/acs.accounts.0c00628. 33242953

[ref3] Chen C. Designing Catalysts for Olefin Polymerization and Copolymerization: Beyond Electronic and Steric Tuning. Nat. Rev. Chem. 2018, 2 (5), 6–14. 10.1038/s41570-018-0003-0.

[ref4] Tan C.; Chen C. Emerging Palladium and Nickel Catalysts for Copolymerization of Olefins with Polar Monomers. Angew. Chem., Int. Ed. 2019, 58 (22), 7192–7200. 10.1002/anie.201814634. 30719812

[ref5] Keyes A.; Basbug Alhan H. E.; Ordonez E.; Ha U.; Beezer D. B.; Dau H.; Liu Y. S.; Tsogtgerel E.; Jones G. R.; Harth E. Olefins and Vinyl Polar Monomers: Bridging the Gap for Next Generation Materials. Angew. Chem., Int. Ed. 2019, 58 (36), 12370–12391. 10.1002/anie.201900650. 30791191

[ref6] Johnson L. K.; Mecking S.; Brookhart M. Copolymerization of Ethylene and Propylene with Functionalized Vinyl Monomers by Palladium(II) Catalysts. J. Am. Chem. Soc. 1996, 118 (1), 267–268. 10.1021/ja953247i.

[ref7] Younkin T. R.; Connor E. F.; Henderson J. I.; Friedrich S. K.; Grubbs R. H.; Bansleben D. A. Neutral, Single-Component Nickel (II) Polyolefin Catalysts That Tolerate Heteroatoms. Science 2000, 287 (5452), 460–462. 10.1126/science.287.5452.460. 10642541

[ref8] Connor E. F.; Younkin T. R.; Henderson J. I.; Hwang S.; Grubbs R. H.; Roberts W. P.; Litzau J. J. Linear Functionalized Polyethylene Prepared with Highly Active Neutral Ni(II) Complexes. J. Polym. Sci., Part A: Polym. Chem. 2002, 40 (16), 2842–2854. 10.1002/pola.10370.

[ref9] Radlauer M. R.; Buckley A. K.; Henling L. M.; Agapie T. Bimetallic Coordination Insertion Polymerization of Unprotected Polar Monomers: Copolymerization of Amino Olefins and Ethylene by Dinickel Bisphenoxyiminato Catalysts. J. Am. Chem. Soc. 2013, 135 (10), 3784–3787. 10.1021/ja4004816. 23425209

[ref10] Takeuchi D.; Chiba Y.; Takano S.; Osakada K. Double-Decker-Type Dinuclear Nickel Catalyst for Olefin Polymerization: Efficient Incorporation of Functional Co-Monomers. Angew. Chem., Int. Ed. 2013, 52 (48), 12536–12540. 10.1002/anie.201307741. 24249551

[ref11] Weberski M. P.; Chen C.; Delferro M.; Marks T. J. Ligand Steric and Fluoroalkyl Substituent Effects on Enchainment Cooperativity and Stability in Bimetallic Nickel(II) Polymerization Catalysts. Chem. – A Eur. J. 2012, 18 (34), 10715–10732. 10.1002/chem.201200713. 22807059

[ref12] Drent E.; Van Dijk R.; Van Ginkel R.; Van Oort B.; Pugh R. I. Palladium Catalysed Copolymerisation of Ethene with Alkylacrylates: Polar Comonomer Built into the Linear Polymer Chain. Chem. Commun. 2002, 2 (7), 744–745. 10.1039/b111252j. 12119702

[ref13] Carrow B. P.; Nozaki K. Synthesis of Functional Polyolefins Using Cationic Bisphosphine Monoxide-Palladium Complexes. J. Am. Chem. Soc. 2012, 134 (21), 8802–8805. 10.1021/ja303507t. 22591442

[ref14] Contrella N. D.; Sampson J. R.; Jordan R. F. Copolymerization of Ethylene and Methyl Acrylate by Cationic Palladium Catalysts That Contain Phosphine-Diethyl Phosphonate Ancillary Ligands. Organometallics 2014, 33 (13), 3546–3555. 10.1021/om5004489.

[ref15] Sui X.; Dai S.; Chen C. Ethylene Polymerization and Copolymerization with Polar Monomers by Cationic Phosphine Phosphonic Amide Palladium Complexes. ACS Catal. 2015, 5 (10), 5932–5937. 10.1021/acscatal.5b01490.

[ref16] Zhang W.; Waddell P. M.; Tiedemann M. A.; Padilla C. E.; Mei J.; Chen L.; Carrow B. P. Electron-Rich Metal Cations Enable Synthesis of High Molecular Weight, Linear Functional Polyethylenes. J. Am. Chem. Soc. 2018, 140 (28), 8841–8850. 10.1021/jacs.8b04712. 29944349

[ref17] Chen M.; Chen C. A Versatile Ligand Platform for Palladium- and Nickel-Catalyzed Ethylene Copolymerization with Polar Monomers. Angew. Chem., Int. Ed. 2018, 57 (12), 3094–3098. 10.1002/anie.201711753. 29372606

[ref18] Mitsushige Y.; Yasuda H.; Carrow B. P.; Ito S.; Kobayashi M.; Tayano T.; Watanabe Y.; Okuno Y.; Hayashi S.; Kuroda J.; Okumura Y.; Nozaki K. Methylene-Bridged Bisphosphine Monoxide Ligands for Palladium-Catalyzed Copolymerization of Ethylene and Polar Monomers. ACS Macro Lett. 2018, 7 (3), 305–311. 10.1021/acsmacrolett.8b00034. 35632905

[ref19] Hong C.; Sui X.; Li Z.; Pang W.; Chen M. Phosphine Phosphonic Amide Nickel Catalyzed Ethylene Polymerization and Copolymerization with Polar Monomers. Dalt. Trans. 2018, 47 (25), 8264–8267. 10.1039/C8DT01018H. 29888779

[ref20] Xin B. S.; Sato N.; Tanna A.; Oishi Y.; Konishi Y.; Shimizu F. Nickel Catalyzed Copolymerization of Ethylene and Alkyl Acrylates. J. Am. Chem. Soc. 2017, 139 (10), 3611–3614. 10.1021/jacs.6b13051. 28218852

[ref21] Fu X.; Zhang L.; Tanaka R.; Shiono T.; Cai Z. Highly Robust Nickel Catalysts Containing Anilinonaphthoquinone Ligand for Copolymerization of Ethylene and Polar Monomers. Macromolecules 2017, 50 (23), 9216–9221. 10.1021/acs.macromol.7b01947.

[ref22] Gao J.; Yang B.; Chen C. Sterics versus Electronics: Imine/Phosphine-Oxide-Based Nickel Catalysts for Ethylene Polymerization and Copolymerization. J. Catal. 2019, 369, 233–238. 10.1016/j.jcat.2018.11.007.

[ref23] Zhang R. F.; Hou Y. H.; Wei X. L.; Zhao D. D.; Cui M. M.; Zhai F. F.; Li X. L.; Liu B. Y.; Yang M. Thermostable α-Diimine Nickel Complexes with Substituents on Acenaphthequinone-Backbone for Ethylene Polymerization. Chin. J. Polym. Sci. 2020, 38 (11), 1214–1220. 10.1007/s10118-020-2430-x.

[ref24] Ito S.; Ota Y.; Nozaki K. Ethylene/Allyl Monomer Cooligomerization by Nickel/Phosphine–Sulfonate Catalysts. Dalt. Trans. 2012, 41 (45), 13807–13809. 10.1039/c2dt31771k. 23059906

[ref25] Chen M.; Chen C. Rational Design of High-Performance Phosphine Sulfonate Nickel Catalysts for Ethylene Polymerization and Copolymerization with Polar Monomers. ACS Catal. 2017, 7 (2), 1308–1312. 10.1021/acscatal.6b03394.

[ref26] Zhang Y.; Mu H.; Pan L.; Wang X.; Li Y. Robust Bulky [P,O] Neutral Nickel Catalysts for Copolymerization of Ethylene with Polar Vinyl Monomers. ACS Catal. 2018, 8 (7), 5963–5976. 10.1021/acscatal.8b01088.

[ref27] Xiong S.; Shoshani M. M.; Zhang X.; Spinney H. A.; Nett A. J.; Henderson B. S.; Miller T. F.; Agapie T. Efficient Copolymerization of Acrylate and Ethylene with Neutral P, O-Chelated Nickel Catalysts: Mechanistic Investigations of Monomer Insertion and Chelate Formation. J. Am. Chem. Soc. 2021, 143 (17), 6516–6527. 10.1021/jacs.1c00566. 33885285

[ref28] Shoshani M. M.; Xiong S.; Lawniczak J. J.; Zhang X.; Miller T. F.; Agapie T. Phosphine-Phenoxide Nickel Catalysts for Ethylene/Acrylate Copolymerization: Olefin Coordination and Complex Isomerization Studies Relevant to the Mechanism of Catalysis. Organometallics 2022, 41 (15), 2119–2131. 10.1021/acs.organomet.2c00241.

[ref29] Conley M. P.; Jordan R. F. Cis/Trans Isomerization of Phosphinesulfonate Palladium(II) Complexes. Angew. Chem., Int. Ed. 2011, 50 (16), 3744–3746. 10.1002/anie.201100065. 21413110

[ref30] Nakano R.; Chung L. W.; Watanabe Y.; Okuno Y.; Okumura Y.; Ito S.; Morokuma K.; Nozaki K. Elucidating the Key Role of Phosphine-Sulfonate Ligands in Palladium-Catalyzed Ethylene Polymerization: Effect of Ligand Structure on the Molecular Weight and Linearity of Polyethylene. ACS Catal. 2016, 6 (9), 6101–6113. 10.1021/acscatal.6b00911.

[ref31] Haras A.; Anderson G. D. W.; Michalak A.; Rieger B.; Ziegler T. Computational Insight into Catalytic Control of Poly(Ethylene - Methyl Acrylate) Topology. Organometallics 2006, 25 (19), 4491–4497. 10.1021/om060250+.

[ref32] Zhou X.; Lau K. C.; Petro B. J.; Jordan R. F. Cis/Trans Isomerization of o -Phosphino-Arenesulfonate Palladium Methyl Complexes. Organometallics 2014, 33 (24), 7209–7214. 10.1021/om501007q.

[ref33] Noda S.; Nakamura A.; Kochi T.; Chung L. W.; Morokuma K.; Nozaki K. Mechanistic Studies on the Formation of Linear Polyethylene Chain Catalyzed by Palladium Phosphine–Sulfonate Complexes: Experiment and Theoretical Studies. J. Am. Chem. Soc. 2009, 131 (39), 14088–14100. 10.1021/ja9047398. 19746977

[ref34] Marenich A. V.; Cramer C. J.; Truhlar D. G. Universal Solvation Model Based on Solute Electron Density and on a Continuum Model of the Solvent Defined by the Bulk Dielectric Constant and Atomic Surface Tensions. J. Phys. Chem. B 2009, 113 (18), 6378–6396. 10.1021/jp810292n. 19366259

[ref35] Zhao Y.; Truhlar D. G. The M06 Suite of Density Functionals for Main Group Thermochemistry, Thermochemical Kinetics, Noncovalent Interactions, Excited States, and Transition Elements: Two New Functionals and Systematic Testing of Four M06-Class Functionals and 12 Other Function. Theor. Chem. Acc. 2008, 120 (1), 215–241. 10.1007/s00214-007-0310-x.

[ref36] Weigend F. Accurate Coulomb-Fitting Basis Sets for H to Rn. Phys. Chem. Chem. Phys. 2006, 8 (9), 1057–1065. 10.1039/b515623h. 16633586

[ref37] Weigend F.; Ahlrichs R. Balanced Basis Sets of Split Valence, Triple Zeta Valence and Quadruple Zeta Valence Quality for H to Rn: Design and Assessment of Accuracy. Phys. Chem. Chem. Phys. 2005, 7 (18), 3297–3305. 10.1039/b508541a. 16240044

[ref38] Goodman J. M.; Silva M. A. QRC: A Rapid Method for Connecting Transition Structures to Reactants in the Computational Analysis of Organic Reactivity. Tetrahedron Lett. 2003, 44 (45), 8233–8236. 10.1016/j.tetlet.2003.09.074.

[ref39] Contreras-García J.; Johnson E. R.; Keinan S.; Chaudret R.; Piquemal J. P.; Beratan D. N.; Yang W. NCIPLOT: A Program for Plotting Noncovalent Interaction Regions. J. Chem. Theory Comput. 2011, 7 (3), 625–632. 10.1021/ct100641a. 21516178 PMC3080048

[ref40] Frisch M. J.; Trucks G. W.; Schlegel H. B.; Scuseria G. E.; Robb M. A.; Cheeseman J. R.; Scalmani G.; Barone V.; Petersson G. A.; Nakatsuji H., Gaussian 16, Revision B.01. Gaussian Inc.2016.

[ref41] Hu L.; Chen H. Assessment of DFT Methods for Computing Activation Energies of Mo/W-Mediated Reactions. J. Chem. Theory Comput. 2015, 11 (10), 4601–4614. 10.1021/acs.jctc.5b00373. 26574251

[ref42] Sun Y.; Chen H. Performance of Density Functionals for Activation Energies of Re-Catalyzed Organic Reactions. J. Chem. Theory Comput. 2014, 10 (2), 579–588. 10.1021/ct4010855. 26580034

[ref43] Sun Y.; Chen H. Performance of Density Functionals for Activation Energies of Zr-Mediated Reactions. J. Chem. Theory Comput. 2013, 9 (11), 4735–4743. 10.1021/ct400432x. 26583392

[ref44] Yu H. S.; He X.; Li S. L.; Truhlar D. G. MN15: A Kohn–Sham Global-Hybrid Exchange–Correlation Density Functional with Broad Accuracy for Multi-Reference and Single-Reference Systems and Noncovalent Interactions. Chem. Sci. 2016, 7 (8), 5032–5051. 10.1039/C6SC00705H. 30155154 PMC6018516

[ref45] O’Duill M. L.; Matsuura R.; Wang Y.; Turnbull J. L.; Gurak J. A.; Gao D. W.; Lu G.; Liu P.; Engle K. M. Tridentate Directing Groups Stabilize 6-Membered Palladacycles in Catalytic Alkene Hydrofunctionalization. J. Am. Chem. Soc. 2017, 139 (44), 15576–15579. 10.1021/jacs.7b08383. 28972751 PMC6002750

[ref46] Liu Z.; Wang Y.; Wang Z.; Zeng T.; Liu P.; Engle K. M. Catalytic Intermolecular Carboamination of Unactivated Alkenes via Directed Aminopalladation. J. Am. Chem. Soc. 2017, 139 (32), 11261–11270. 10.1021/jacs.7b06520. 28727452

[ref47] Fukui K. Formulation of the Reaction Coordinate. J. Phys. Chem. 1970, 74 (23), 4161–4163. 10.1021/j100717a029.

[ref48] Fukui K. The Path of Chemical Reactions - The IRC Approach. Acc. Chem. Res. 1981, 14 (12), 363–368. 10.1021/ar00072a001.

[ref49] Grimme S. Supramolecular Binding Thermodynamics by Dispersion-Corrected Density Functional Theory. Chem. Eur. J. 2012, 18 (32), 9955–9964. 10.1002/chem.201200497. 22782805

[ref50] Luchini G.; Alegre-Requena J. V.; Funes-Ardoiz I.; Paton R. S. GoodVibes: Automated Thermochemistry for Heterogeneous Computational Chemistry Data. F1000research 2020, 9, 291 10.12688/f1000research.22758.1.

[ref51] Bryantsev V. S.; Diallo M. S.; Goddard Iii W. A.; Goddard W. A. Calculation of Solvation Free Energies of Charged Solutes Using Mixed Cluster/Continuum Models. J. Phys. Chem. B 2008, 112 (32), 9709–9719. 10.1021/jp802665d. 18646800

[ref52] Boyle B. T.; Levy J. N.; de Lescure L.; Paton R. S.; McNally A. Halogenation of the 3-Position of Pyridines through Zincke Imine Intermediates. Science 2022, 378 (6621), 773–779. 10.1126/science.add8980. 36395214 PMC10631470

[ref53] Darù A.; Hu X.; Harvey J. N. Iron-Catalyzed Reductive Coupling of Alkyl Iodides with Alkynes to Yield Cis-Olefins: Mechanistic Insights from Computation. ACS Omega 2020, 5 (3), 1586–1594. 10.1021/acsomega.9b03578. 32010833 PMC6990637

[ref54] Guin S.; Dolui P.; Zhang X.; Paul S.; Singh V. K.; Pradhan S.; Chandrashekar H. B.; Anjana S. S.; Paton R. S.; Maiti D. Iterative Arylation of Amino Acids and Aliphatic Amines via δ-C(Sp^3^)–H Activation: Experimental and Computational Exploration. Angew. Chem., Int. Ed. 2019, 58 (17), 5633–5638. 10.1002/anie.201900479. 30821038

[ref55] Glendening E. D.; Reed A. E.; Carpenter J. E.; Weinhold F. Gaussian NBO Version 3.1 Gaussian Inc. 2001

[ref56] Sosa C.; Andzelm J.; Elkin B. C.; Wimmer E.; Dobbs K. D.; Dixon D. A. A Local Density Functional Study of the Structure and Vibrational Frequencies of Molecular Transition-Metal Compounds. J. Phys. Chem. 1992, 96 (16), 6630–6636. 10.1021/j100195a022.

[ref57] Godbout N.; Salahub D. R.; Andzelm J.; Wimmer E. Optimization of Gaussian-Type Basis Sets for Local Spin Density Functional Calculations. Part I. Boron through Neon, Optimization Technique and Validation. Can. J. Chem. 1992, 70 (2), 560–571. 10.1139/v92-079.

[ref58] Schrödinger PyMOL Molecular Graphics Development Component Version 1.8. Schrödinger 2015.

