# Supplementary material for: Unraveling the Mechanism and Influence of Auxiliary Ligands on the Isomerization of Neutral [P,O]-Chelated Nickel Complexes for Olefin Polymerization
Source: J Org Chem. 2025 Jan 28;90(5):2052–61. doi: 10.1021/acs.joc.4c02856 (PMC11811995; doi:10.1021/acs.joc.4c02856)
Supplement: Supplementary file 1 — jo4c02856_si_001.pdf [file jo4c02856_si_001.pdf]

Electronic Supporting Information for

Unravelling the Mechanism and Influence of Auxiliary Ligands on the Isomerization of Neutral [P,O]-Chelated Nickel Complexes for Olefin Polymerization

Jeremy Tan,[c,d] Jingyi Liu,[a,d] and Xinglong Zhang*,[a,b]

[a] Prof. Dr. X. Zhang, J. Liu

Department of Chemistry, The Chinese University of Hong Kong, Shatin, New Territories, Hong Kong, China

* Email: xinglong.zhang@cuhk.edu.hk

[b] Dr. X. Zhang

Institute of High Performance Computing, Agency for Science, Technology and Research (A*STAR), 1 Fusionopolis Way, #16-16 Connexis, Singapore, 138632, Republic of Singapore

[c] J. Tan

Department of Chemistry, National University of Singapore, 4 Science Drive 2, Singapore, 117544, Republic of Singapore

[d] These authors contributed equally.

Table of Contents

The Computational Section S2

1. *Conformational considerations* S3
2. *Co-monomer Insertion potential energy surface (PES)* S3
3. *Catalyst XIII (POP-Ni-py)* S5
   - 3.1 **Relaxed potential energy surface (PES) scan for dissociation of pyridine** S5
   - 3.2 **DFT optimized geometries** S5
   - 3.3 **Frontier Molecular Orbitals** S7
4. *Catalyst XIV (PONap-Ni-py)* S9
   - 4.1 **Stepwise pseudorotation** S9
5. *Binding energies calculations* S11
6. *Effect of auxiliary ligands on isomerization barrier* S13
7. *Determination of competing chemical reactivity using simple transition state theory* ..S29
8. *Absolute contribution to Gibbs energies* S29
9. *Optimised geometries* S32
10. *References* S33

The Computational Section

Geometry optimizations were performed using hybrid meta-generalized gradient-approximation (hybrid meta-GGA) M06 functional[35] with Karlsruhe-family basis set of double-ζ valence def2-SVP[36,37] for all atoms with implicit SMD continuum solvation model[34] was used to account for the solvent effect of chlorobenzene as implemented in *Gaussian 16* rev. B.01.[40] Where possible, available X-ray crystal structures were used as an initial guess. The M06 functional was chosen as it performs better than many other functionals (e.g. ωB97X-D and TPSS) in predicting transition metal (TM) reaction barrier heights (TMBH21 dataset[41–43]) for reactions involving TMs.[35,44] M06 has also been employed to study similar TM-catalyzed systems with excellent agreement with experimental results.[45,46] Minima and transition structures on the potential energy surface (PES) were confirmed as such by harmonic frequency analysis, showing respectively zero and one imaginary frequency, at the same level of theory. Where appropriate, intrinsic reaction coordinate (IRC) analyses[47,48] were performed to confirm that the found TSs connect to the right reactants and products.

Single point (SP) corrections were performed using M06 functional and def2-TZVP[37] basis set for all atoms. The implicit SMD continuum solvation model[34] was used to account for the solvent effect of chlorobenzene on the Gibbs energy profile. Gibbs energies were evaluated at the reaction temperature of 323.15 K, using Grimme's scheme of quasi-RRHO treatment of vibrational entropies[49], using the GoodVibes code[50]. Vibrational entropies of frequencies below 100 cm^{-1} were obtained according to a free rotor description, using a smooth damping function to interpolate between the two limiting descriptions. The free energies reported in Gaussian from gas-phase optimisation were further corrected using standard concentration of 1 mol/L,[51–53] which were used in solvation calculations, instead of the gas-phase 1atm used by default in Gaussian program. SMD(chlorobenzene)-M06/def2-TZVP//SMD(chlorobenzene)-M06/def2-SVP Gibbs energies are given and quoted in kcal mol^{-1} throughout. *Unless otherwise stated, these solvent-corrected values are used for discussion throughout the main text and in this supporting information.*

Natural bond orbital (NBO) analysis and Wiberg bond indices (WBI) calculations, following the example in ref.[54], was performed in Gaussian using NBO version 3.1.[55] Non-covalent interactions (NCIs) were analyzed using NCIPLOT[39] calculations. The *.wfn* files for NCIPLOT were generated at M06/def2-SVP[56,57] level of theory. NCI indices calculated with NCIPLOT were visualized at a gradient isosurface value of $s = 0.5$ au. These are colored according to the sign of the second eigenvalue (λ_2) of the Laplacian of the density ($\nabla^2\rho$) over the range of -0.1 (blue = attractive) to +0.1 (red = repulsive). Molecular orbitals are visualized using an isosurface value of 0.05 throughout. All molecular structures and molecular orbitals were visualized using *PyMOL* software.[58] *Unless otherwise stated, all energy values are quoted in kcal mol^{-1} and bond distances in Å.*

1. Conformational considerations

Where available, experimentally obtained X-ray crystal structures were used as initial guess for geometry optimization. Where different conformers exist in the X-ray structures, all available conformers were used for geometry optimization and the final optimized, lowest energy structure is used. The ligand backbone from the lowest energy conformer is then kept fixed for all subsequent reaction paths.

2. Co-monomer Insertion potential energy surface (PES)

The co-monomer insertion PES is shown in Figure 1 of the main text. The DFT optimized TS structures are shown in Figure S1.

**ts1-et**	**ts1-ac**
$\Delta G^{\ddagger}$ = 32.7	$\Delta G^{\ddagger}$ =30.3

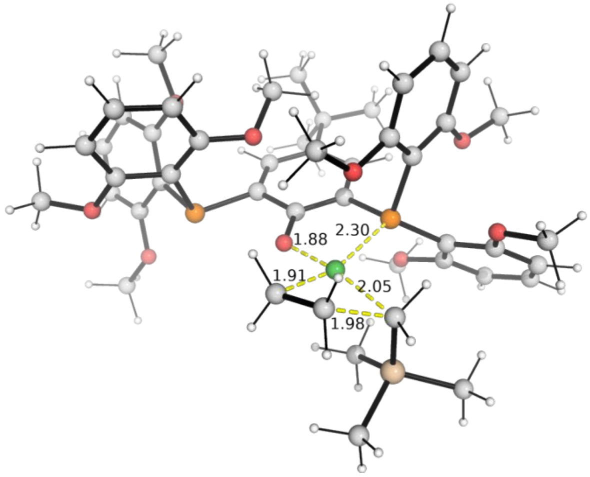	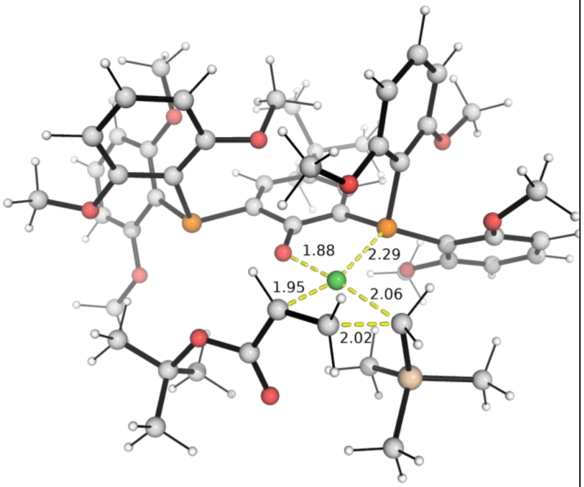
**ts1’-et**	**ts1’-ac**
ΔG‡ = 20.5	ΔG‡ =22.0
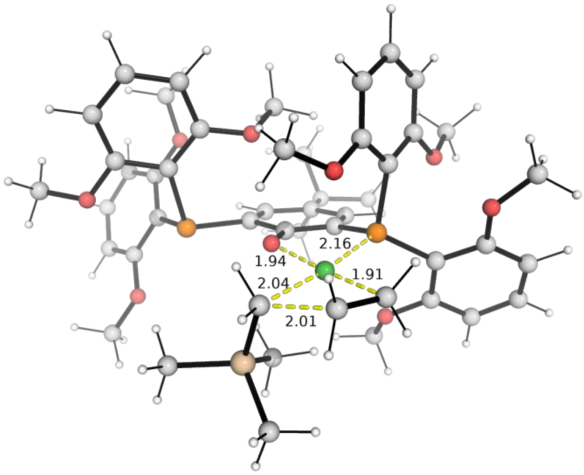	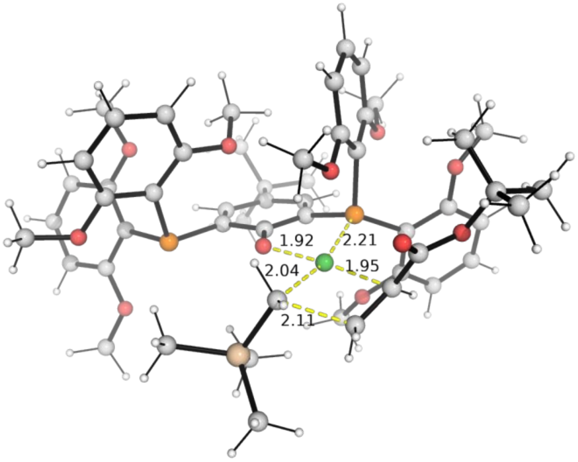

Figure S1. DFT-optimized structures for the monomer insertion step for **A(XIII)L1** and **B(XIII)L1**. The Gibbs energies are calculated relative to lowest energy species **A(XIII)L1**. Gibbs energy values are given in kcal mol^{-1}. Key bond distances are given in Å.

3. Catalyst XIII (POP-Ni-py)

3.1 Relaxed potential energy surface (PES) scan for dissociation of pyridine

The relaxed PES scan for the dissociation of pyridine ligand from the complex **A(XIII)Pyridine** along the Ni–N(pyridine) bond distance is shown in Figure S2. From the Figure, we can see that the dissociation of pyridine from the complex is unfavorable and uphill by about 27 kcal/mol. The optimization using structure 5 in Figure S2 as initial guess results in a complex where pyridine is uncoordinated (**A-1**, Figure S3) and this is uphill by 22.4 kcal/mol. This unfavorability results from the loss of Ni–N enthalpic interaction as pyridine uncoordinates from the Ni-center.

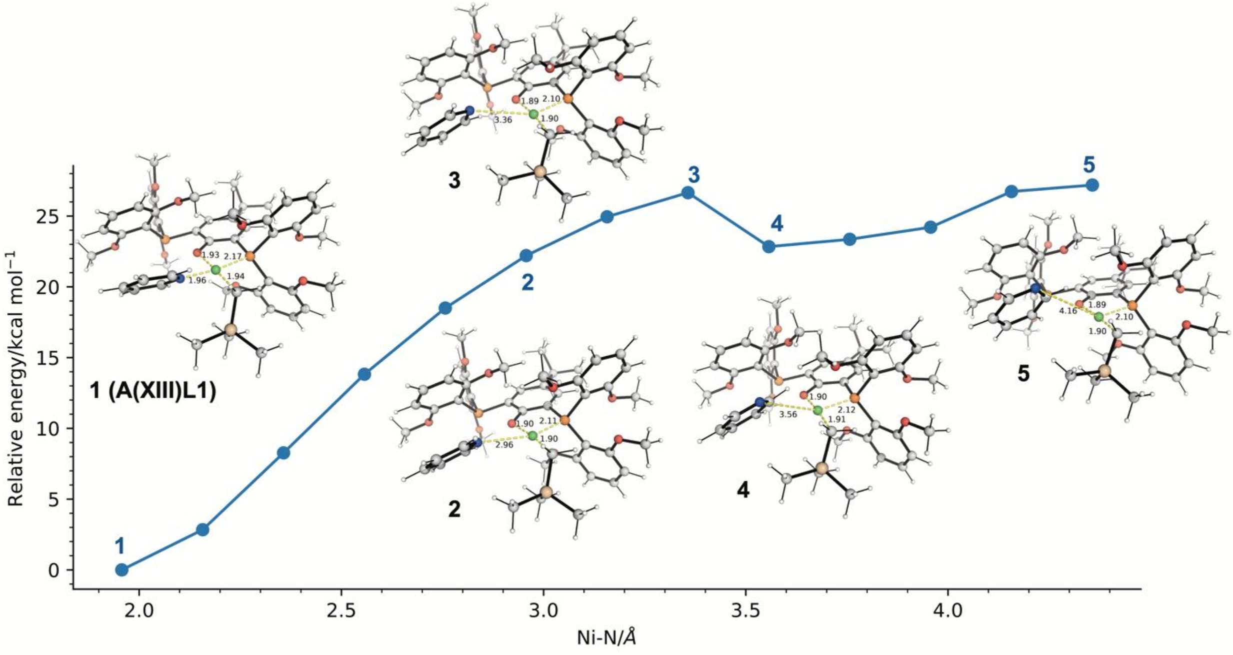

Figure S2. Relaxed PES scan along the Ni–N(pyridine) bond distance. Relative energy values are computed at SMD(chlorobenzene)-M06/def2-SVP level of theory and used without further corrections. Key bond distances are given in Å.

3.2 DFT optimized geometries

The loss of P-coordination replaced by methoxy O-coordination was considered. However, the resulting species, **A1c**, has very high energy such that their formation is highly endergonic and unfavorable (Figure S3).

A-et	A1a
ΔG = 5.9	ΔG = 22.4
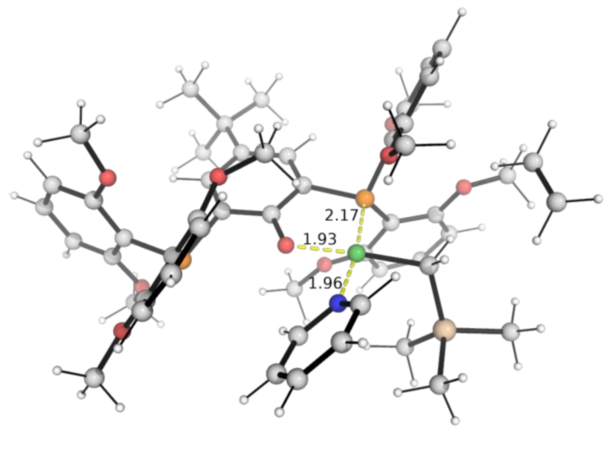	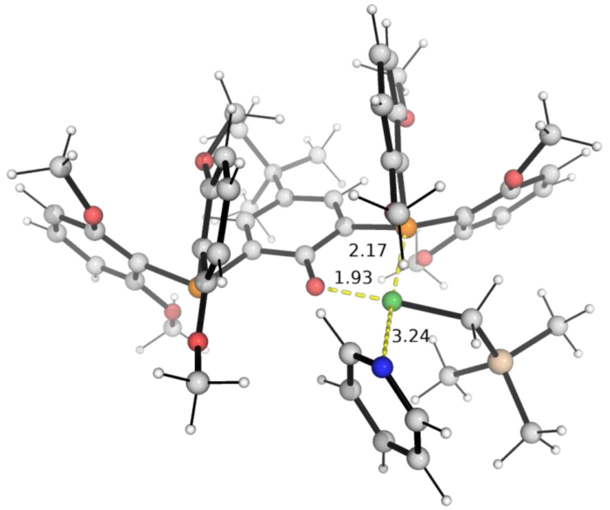
**A1b**	**A1b-c2**
ΔG = 28.7	ΔG = 30.2
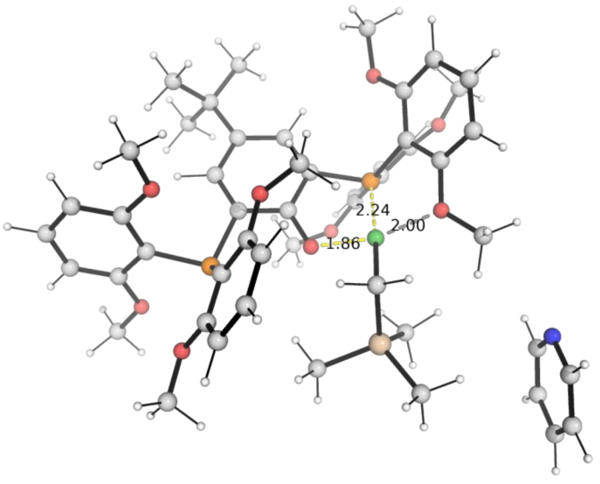	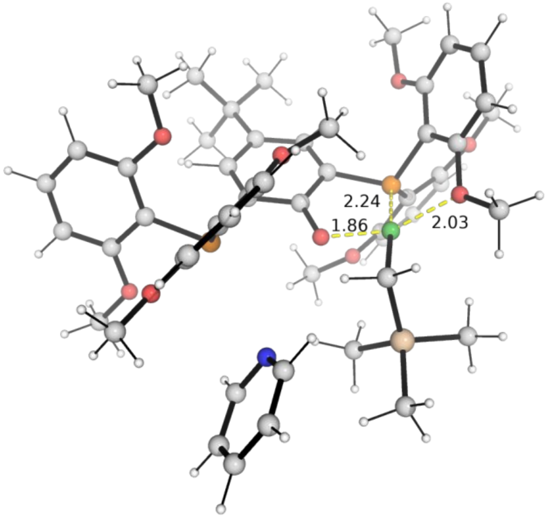
**A1c**	**TS_tet_XIII-c2**
ΔG = 30.2	ΔG‡ = 35.0

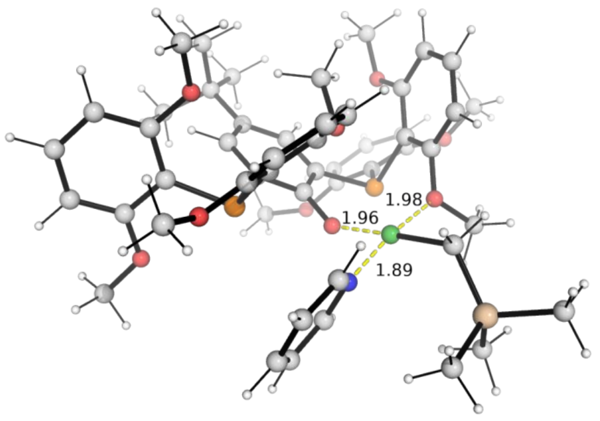	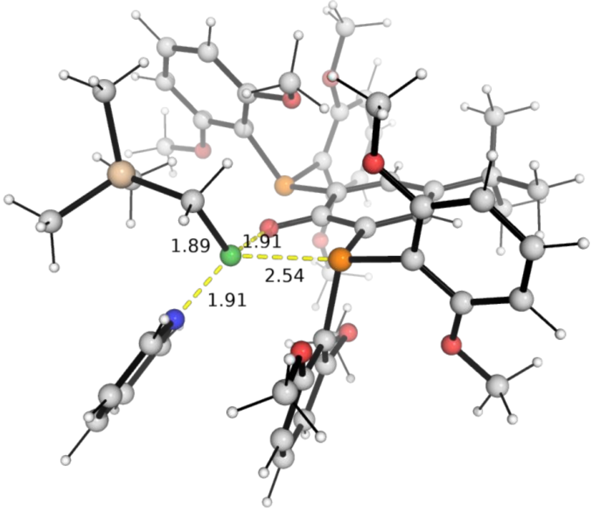
**TS_5coord_XIII-c2**	
ΔG‡ = 28.9	
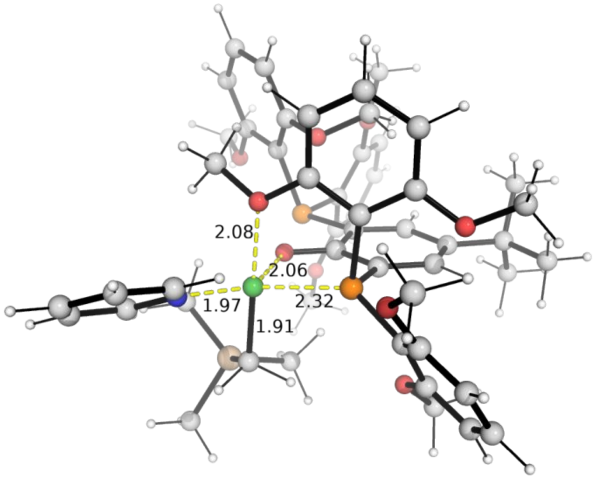	

Figure S3. DFT-optimized structures for the isomerization of **A(XIII)L1** to **B(XIII)L1**. The Gibbs energies are calculated relative to lowest energy species **A(XIII)L1**. Gibbs energy values are given in kcal mol^{-1}. Key bond distances are given in Å.

3.3 Frontier Molecular Orbitals

The frontier molecular orbitals (FMOs) for the competing TSs for the isomerization are shown in Figure S4.

**TS_3coord_XIII**	
HOMO	LUMO
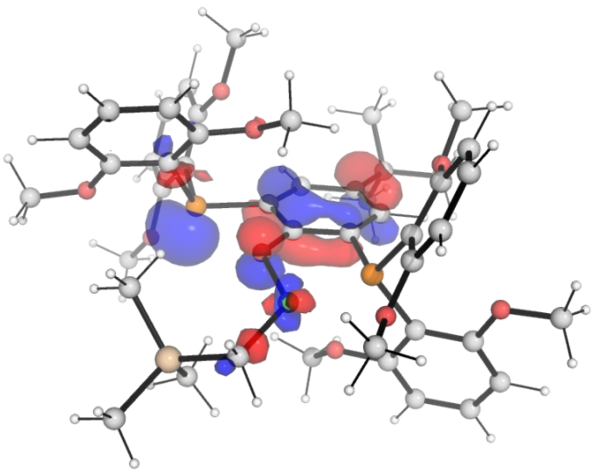	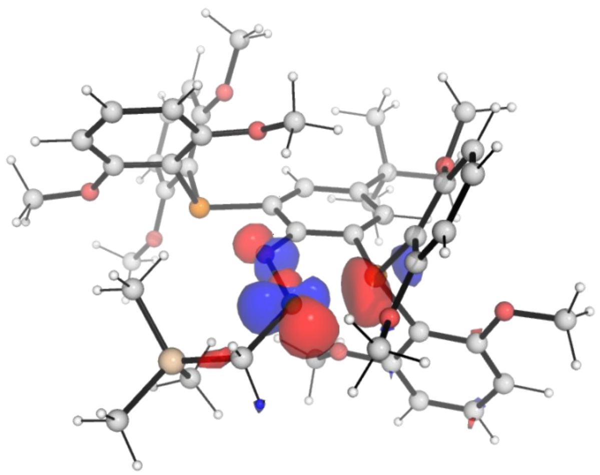
**TS_tet_XIII**	
HOMO	LUMO
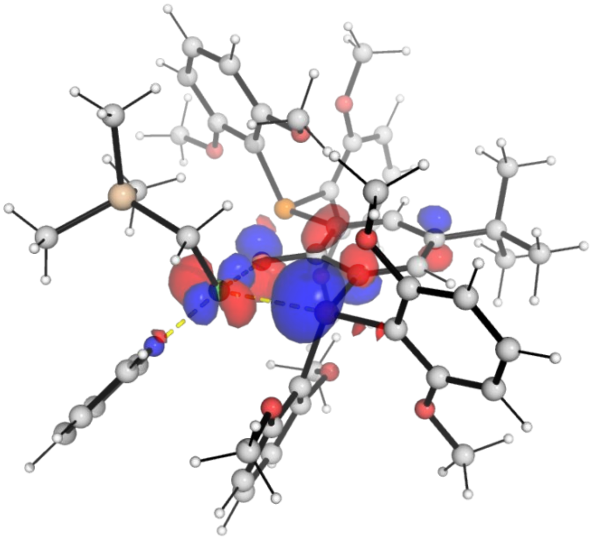	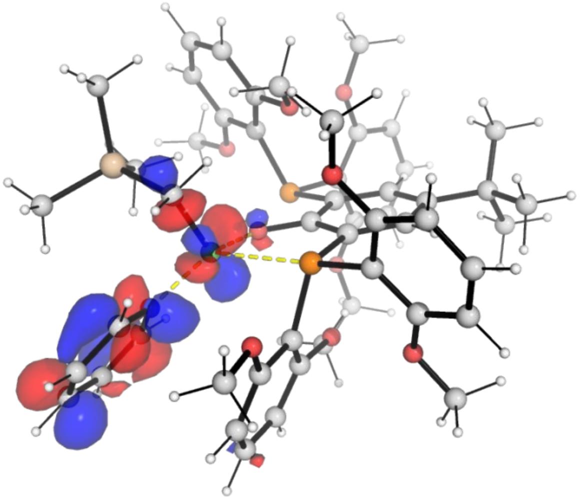
**TS_5coord_XIII**	
HOMO	LUMO

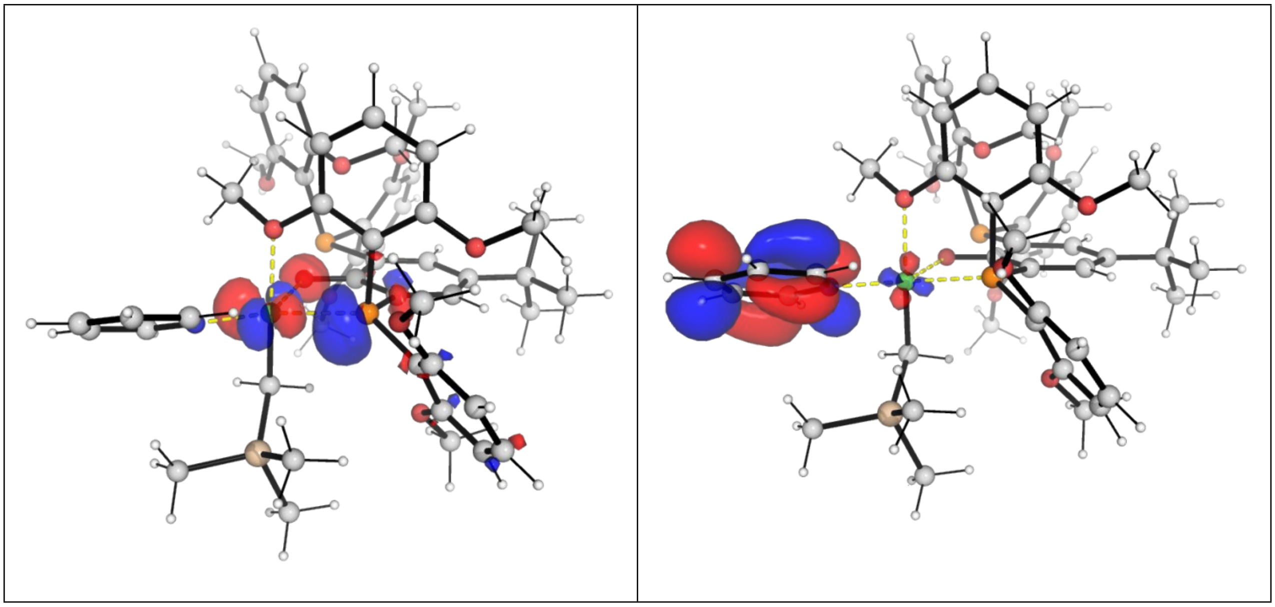

Figure S4. Frontier molecular orbitals (FMOs) for the competing TSs .

4. Catalyst XIV (PONap-Ni-py)

4.1 Stepwise pseudorotation

It is interesting to note that for the isomerization of catalyst XIV via 5-coordinate species, a stepwise mechanism involving two sequential TSs through a stable intermediate, A(XIV)L1 → TS1 → INT → TS2 → B(XIV)L1, was found. The Gibbs energy profile for this process is shown in Figure 3 **of main text** and the DFT optimized structures are shown in Figure S5.

**A(XIV)L1**	**TS1/TS_5coord_XIV**

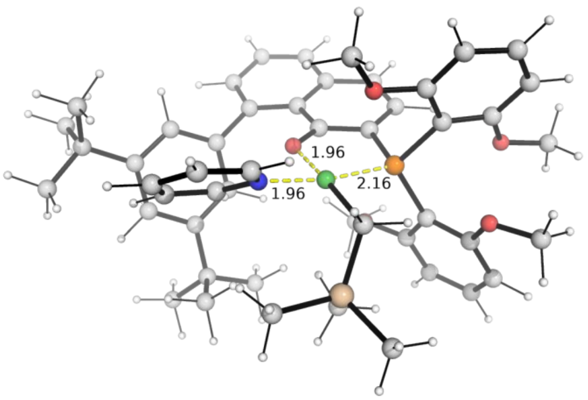

INT

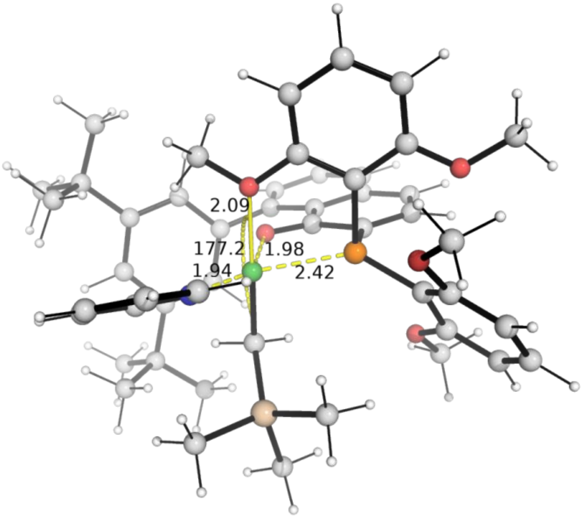

TS2

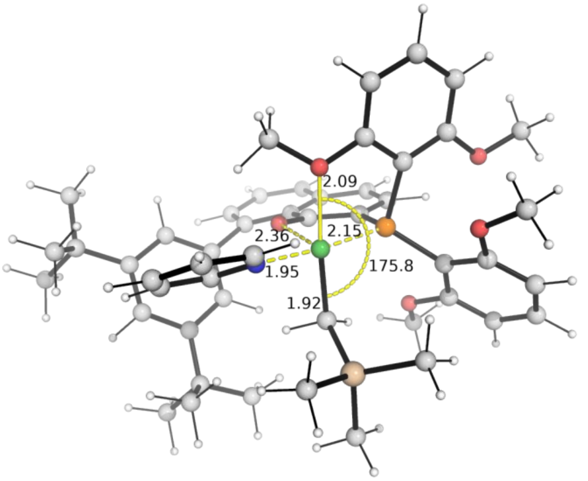

B(XIV)L1

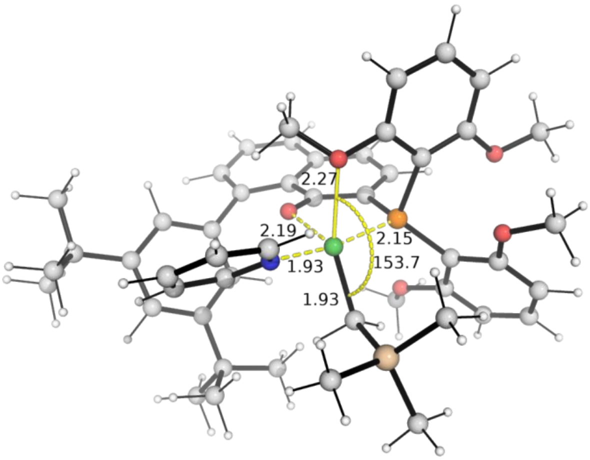

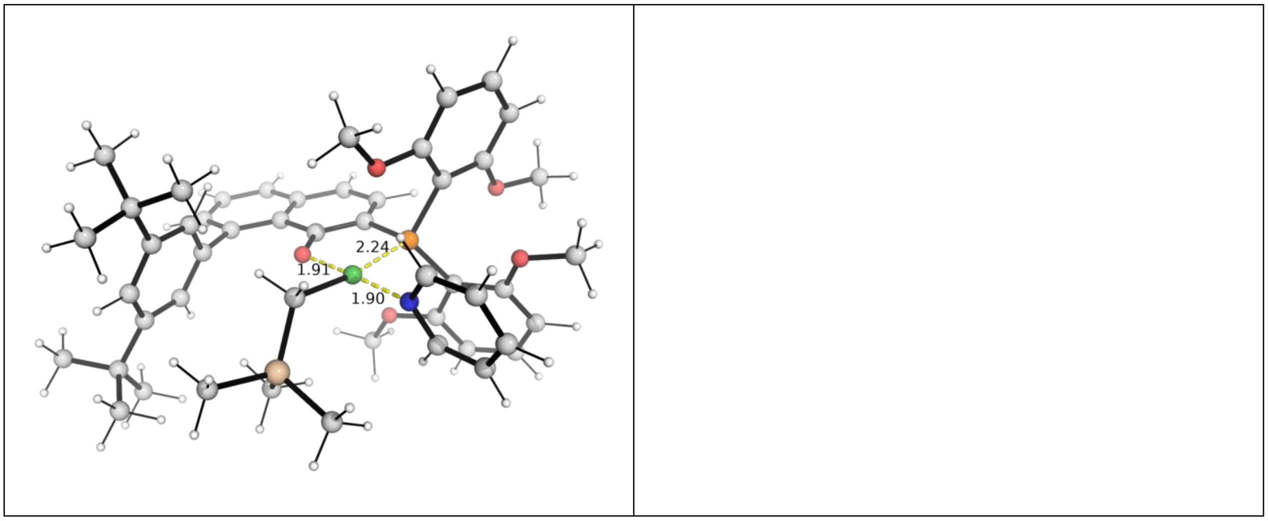

Figure S5. DFT-optimized structures for the two-step isomerization of **A(XIV)L1** to **B(XIV)L1**. Key bond distances are given in Å. Key angles are given in degrees.

5. Binding energies calculations

The Gibbs energy for the binding of ligand **L** is calculated for the reaction shown below:

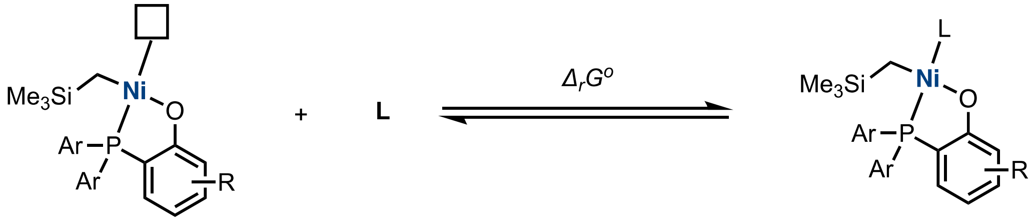

The Gibbs energy of reaction under standard conditions, $\Delta_r G^o$, is related to the equilibrium constant K_{eq} via:

$$\Delta_r G^o = -RT\ln(K_{eq})$$

where R is the universal gas constant and T is the temperature.

Thus, the ratio of equilibrium constants between two paths A and B can be related via,

$$\frac{K_{eq,A}}{K_{eq,B}} = \frac{e^{-\Delta_r G^o_A/RT}}{e^{-\Delta_r G^o_B/RT}} = e^{-\Delta\Delta_r G^o/RT}$$

where $\Delta_r G_X{}^o$ is the Gibbs energy of reaction for path X (X = A, B) and $\Delta\Delta_r G^o = \Delta_r G_A{}^o - \Delta_r G_B{}^o$, is the difference between the Gibbs energies for paths A and B. Thus, on the log scale, we have,

$$\log\left[\frac{K_{eq,A}}{K_{eq,B}}\right] = \log\left[e^{-\Delta\Delta_r G^o/RT}\right]$$

Table S1. Experimentally measured and computed ligand competition values via logarithm of ratio of equilibrium constants.

$\log(K_{L/py})$	L1	L2	L3	L4	L5
**XIII Expt**	0	-1.8	-3.9	0.2	-4.3
**XIII Comp**	0	-1.1	-2.3	-0.1	-7.5
**XIV Expt**	0	-1.8	-4.4	-0.2	-5.1
**XIV Comp**	0	-0.9	-3.6	-1.6	-8.9

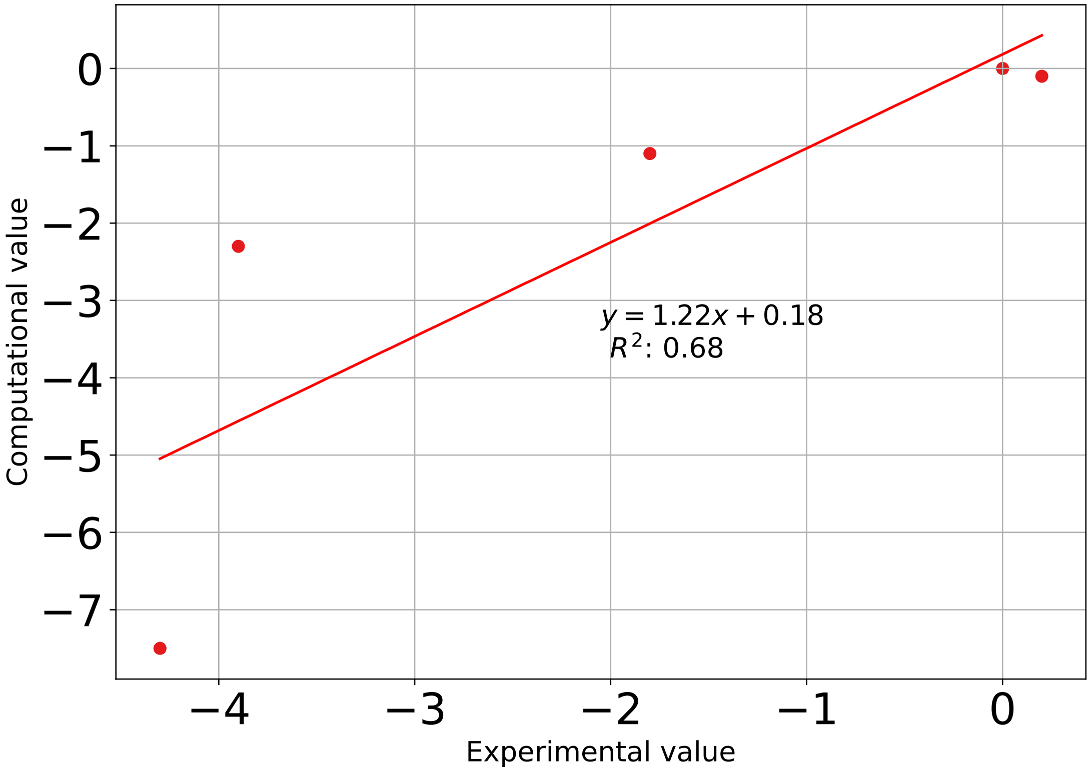

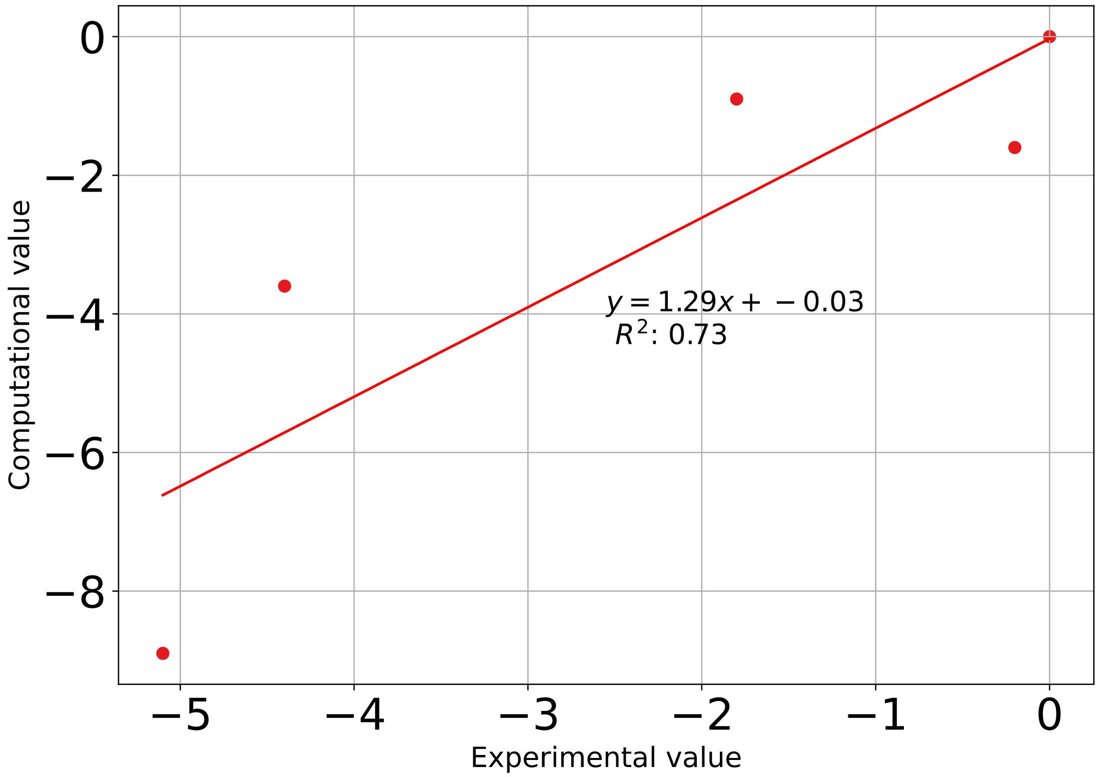

Figure S6. Plot of experimental vs computed values for the log of ratio of equilibrium constants. Top: for catalyst system **XIII**; and bottom: for catalyst system **XIV**.

6. Effect of auxiliary ligands on isomerization barrier

For the TS structures that could not be located, we performed an estimation study using the following method: We took the true TS structure with pyridine ligand (**L1**) bound to the system and modify the ligand structure (pyridine to others; **L1** to **L2–L5**) individually, without changing the rest of the structure. The modified structure is subject to constrained geometry optimization where all atoms, other than the ligand submolecule, are frozen. The DFT structure resulting from this constrained geometry optimization was run at the same level of theory for frequency calculations and higher level single-point correction, as previously to obtain the estimated barrier heights.

Table S2 shows the actual and the estimated values. For each catalyst system with ligand **L1**, this is taken as the reference so that the actual and estimated have the same value. For TSs that we have the true activation barriers, we still performed the estimate calculation to see how good the estimation is. We note that the estimated barriers are all

lower than the actual barriers by 0.6–1.5 kcal/mol, except for catalyst **XIII** with ligand **L2**, where the estimated barrier is 1.3 kcal/mol higher. We note that the estimated barriers show no systematic trend, although the differences fall within ± 2 kcal/mol for the ones we have the true TS barriers.

Table S2. Computed actual vs estimated activation barriers for isomerization. **L2-c2** is the second conformation using **L2** where the methyl group points in the other direction as that in **L2**.

System	L1	L2	L2-c2	L3	L4	L5
**XIII Actual**	28.2	26.0	26.8	–	27.5	–
**XIII Estimated**	28.2	27.3	26.2	29.6	26.1	26.0
**XIV Actual**	24.2	23.5	–	–	22.7	–
**XIV Estimated**	24.2	22.0	–	26.5	21.4	23.5

DFT optimized structures for the true TSs and the constrained DFT optimized structures for estimation of true TS barriers are shown in Figure S7. The frontier molecular orbital plots and non-covalent interaction plots for the true TSs are show in Figure S8.

Catalyst XIII	
**TS_5coord_XIII_L2**	**TS_5coord_XIII_L2-c2**

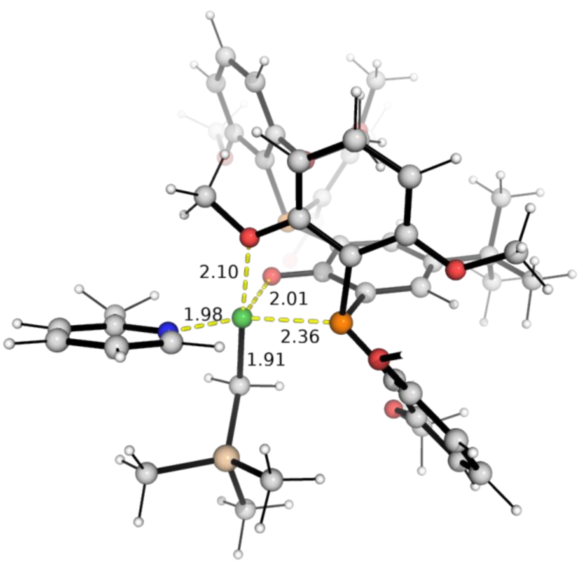

TS_5coord_XIII_L3 (est.)

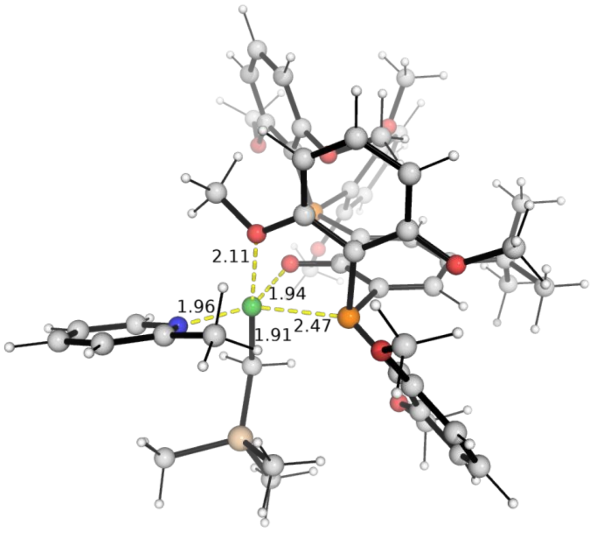

TS_5coord_XIII_L4

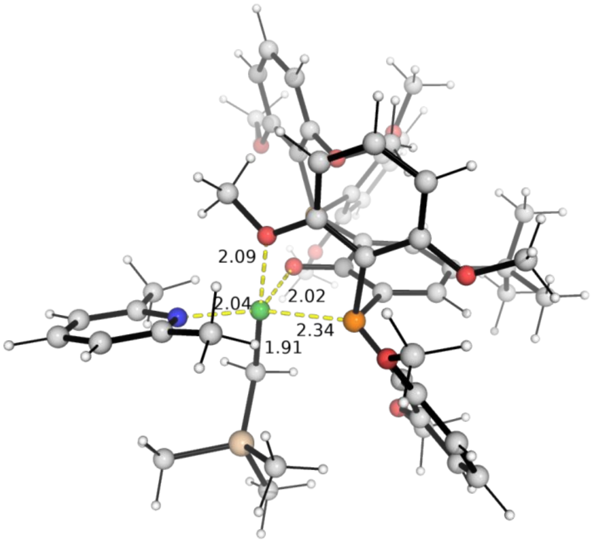

TS_5coord_XIII_L5 (est.)

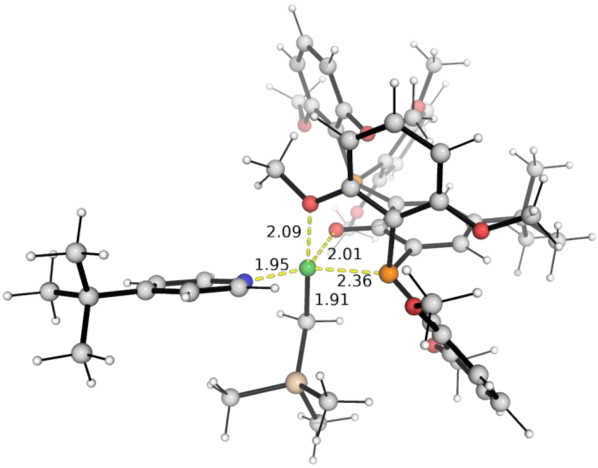

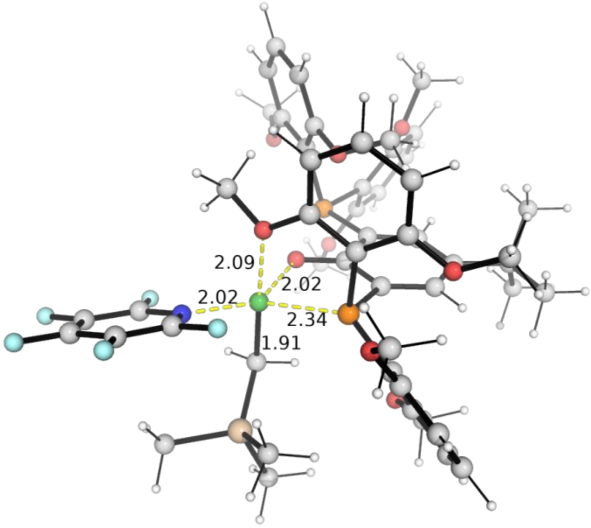	
**Catalsyt XIV**	
**TS_5coord_XIV_L2**	**TS_5coord_XIV_L3 (est.)**
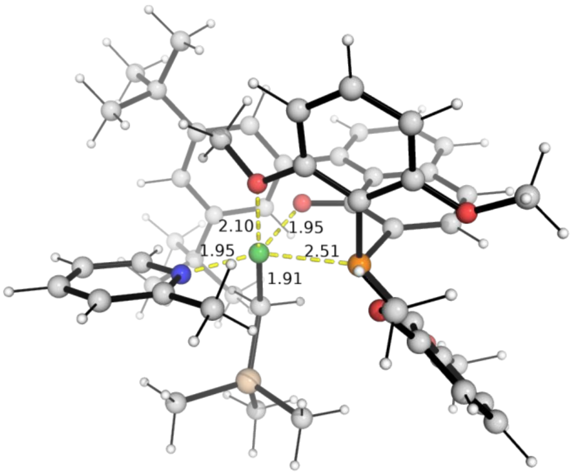	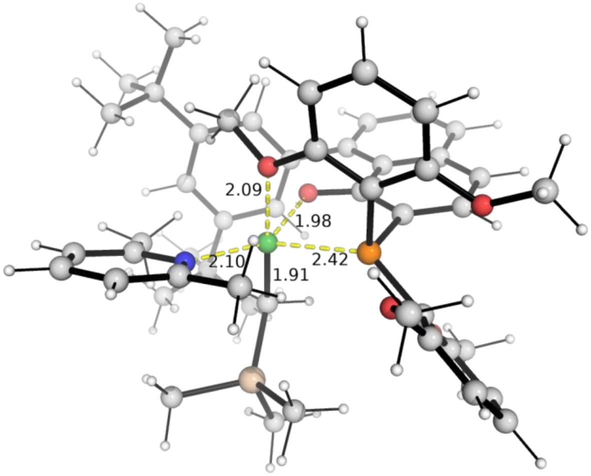
**TS_5coord_XIV_L4**	**TS_5coord_XIV_L5 (est.)**

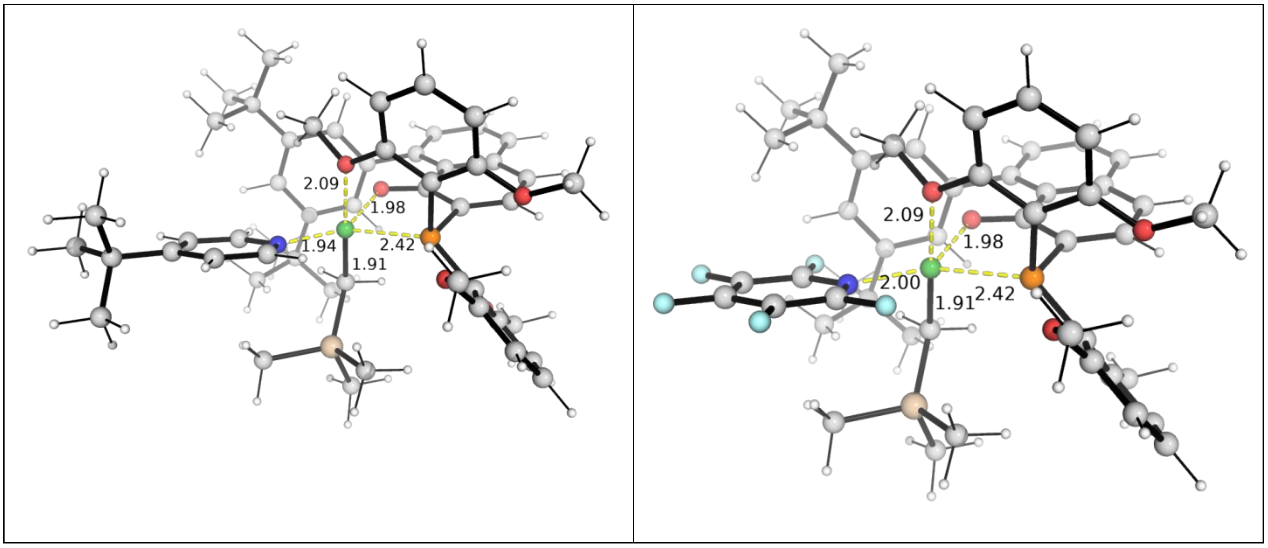

Figure S7. DFT-optimized structures for the TS of isomerization via penta-coordinate mode and constrained optimized structures of the models used for estimation of real TS barrier. The latter is denoted with (est.) inside the bracket. Key bond distances are given in Å. Key angles are given in degrees.

TS XIII	HOMO	LUMO	NCI plot
**L1**	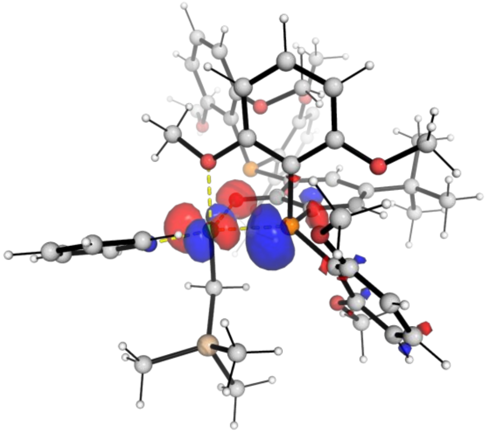	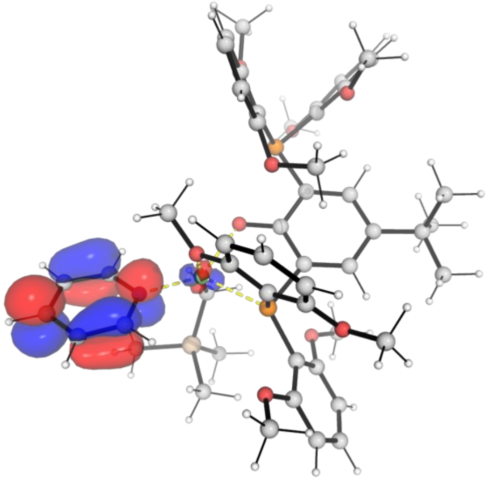	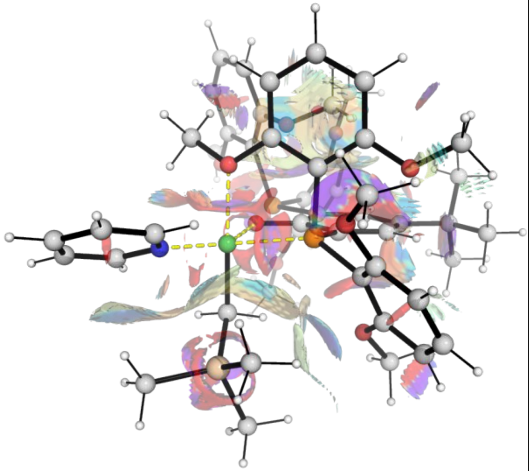
**L2**	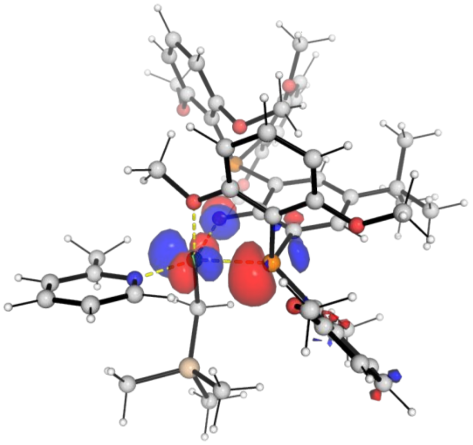	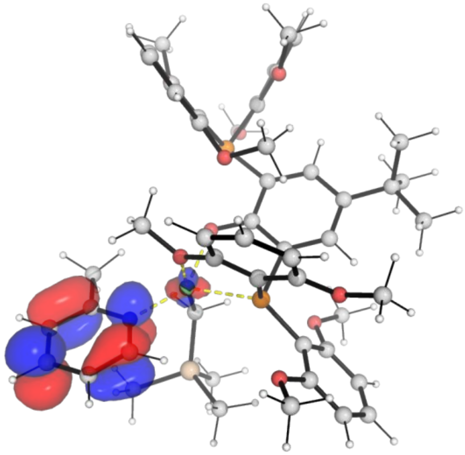	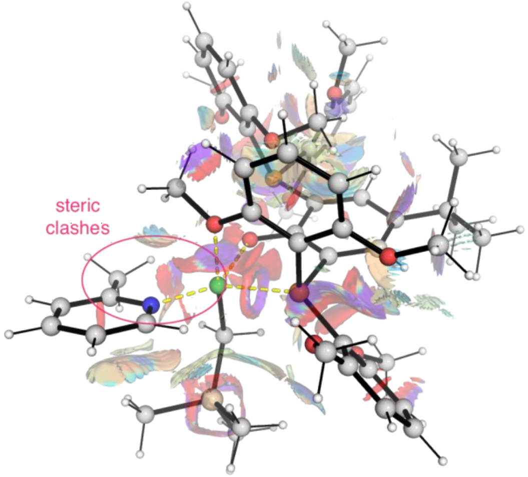

**L3^{est}**	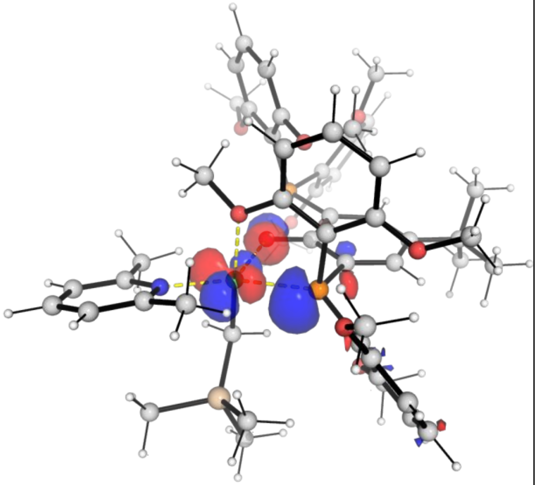	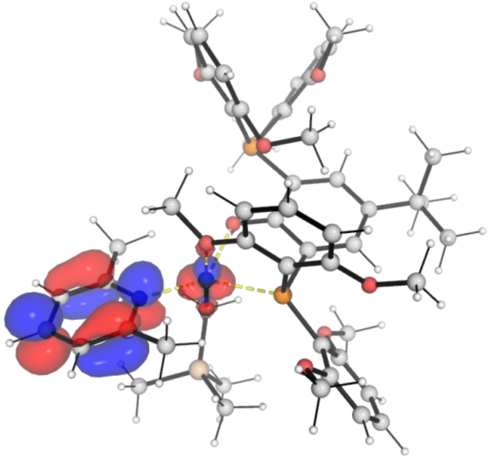	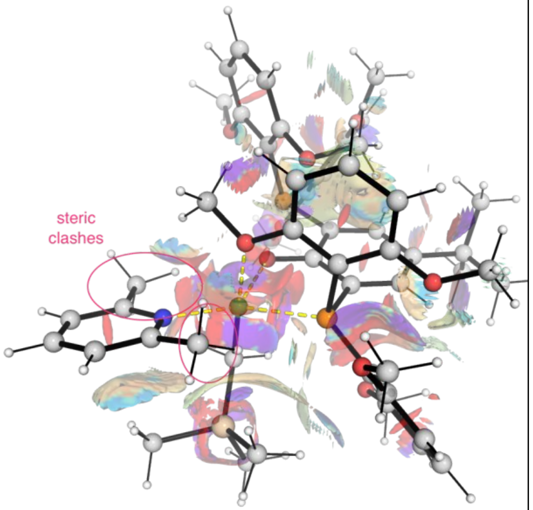
**L4**	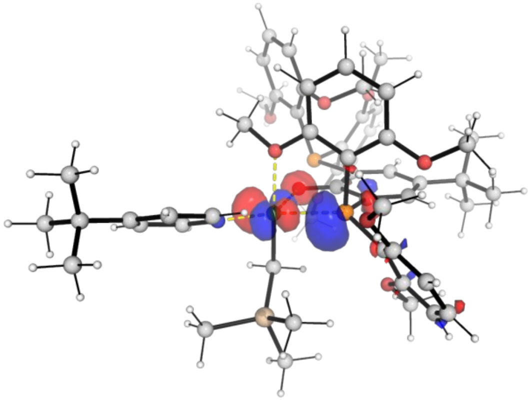	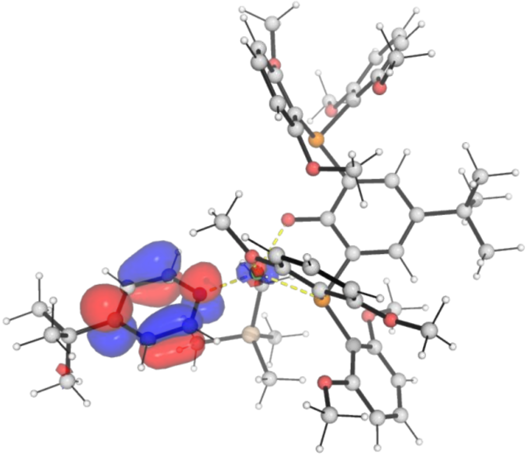	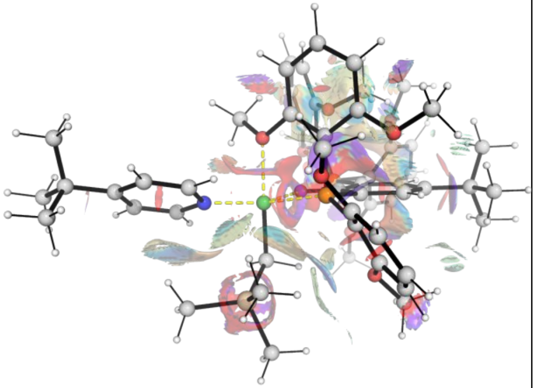

**L5^{est}**	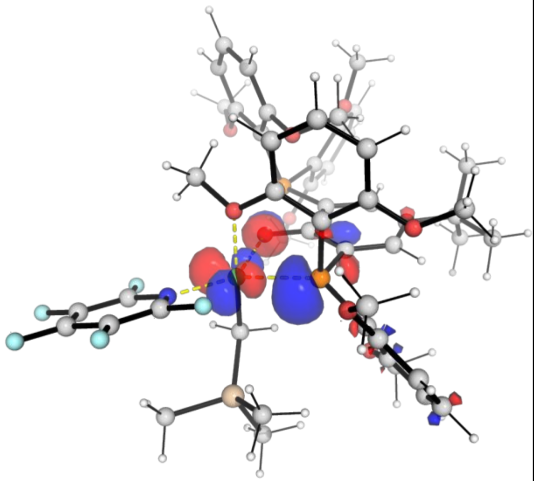	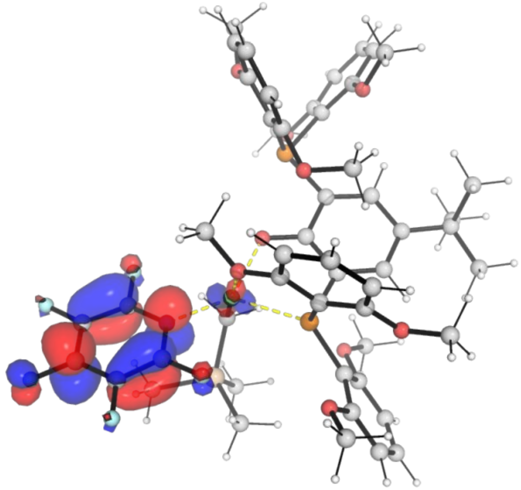	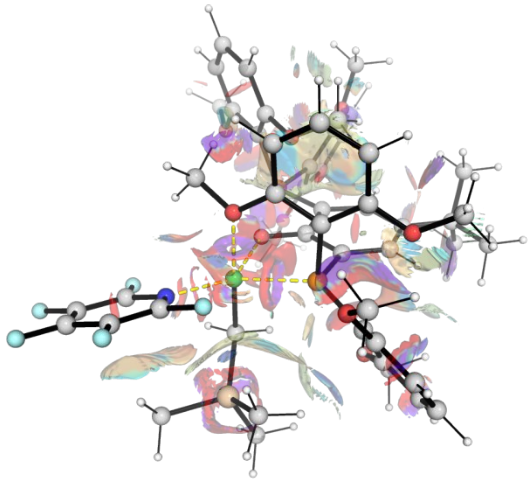
**TS XIV**	**HOMO**	**LUMO**	**NCI plot**
**L1**	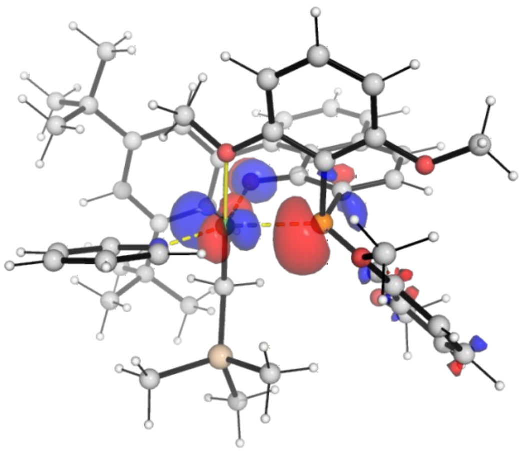	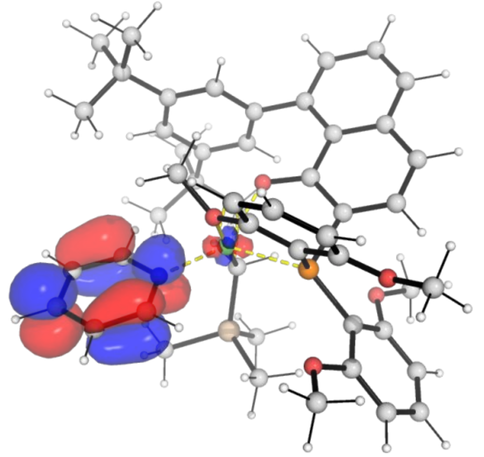	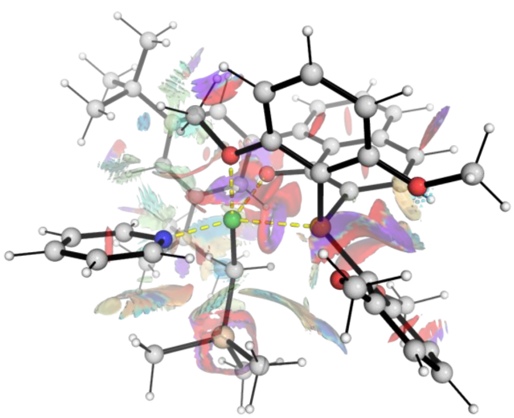

**L2**	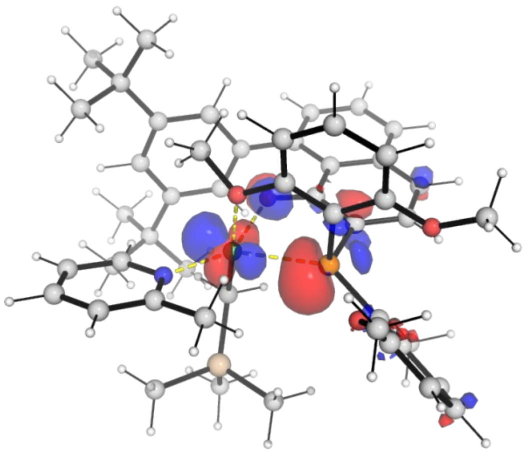	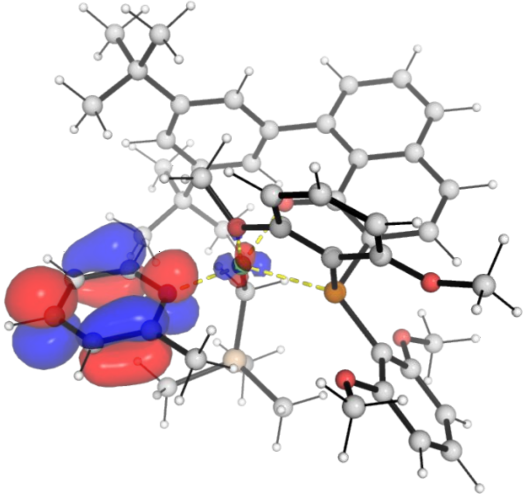	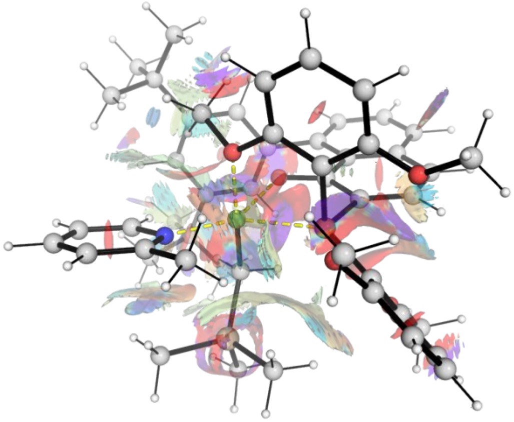
**L3^{est}**	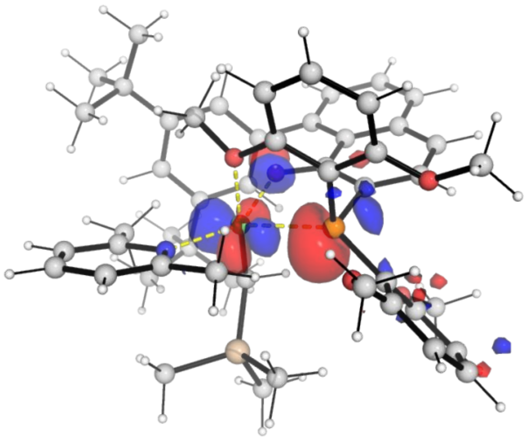	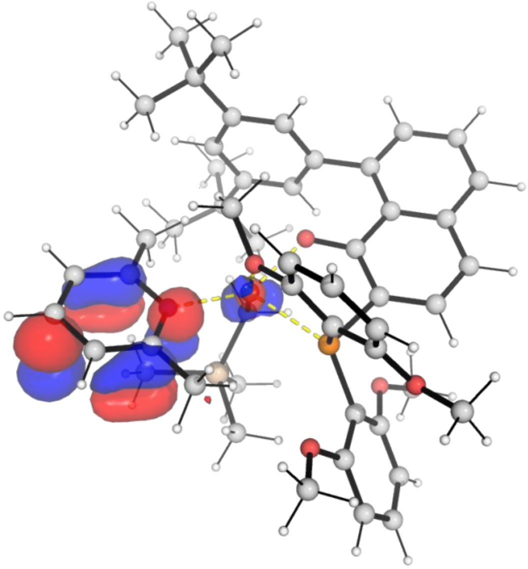	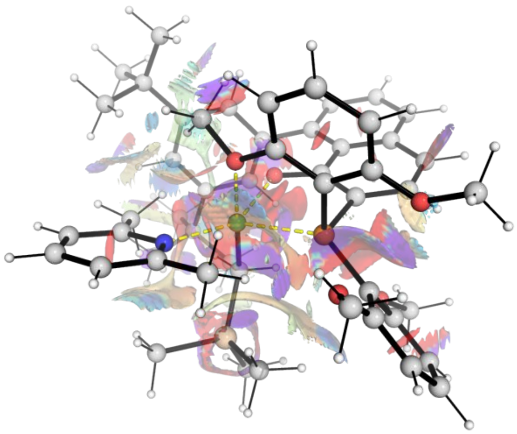

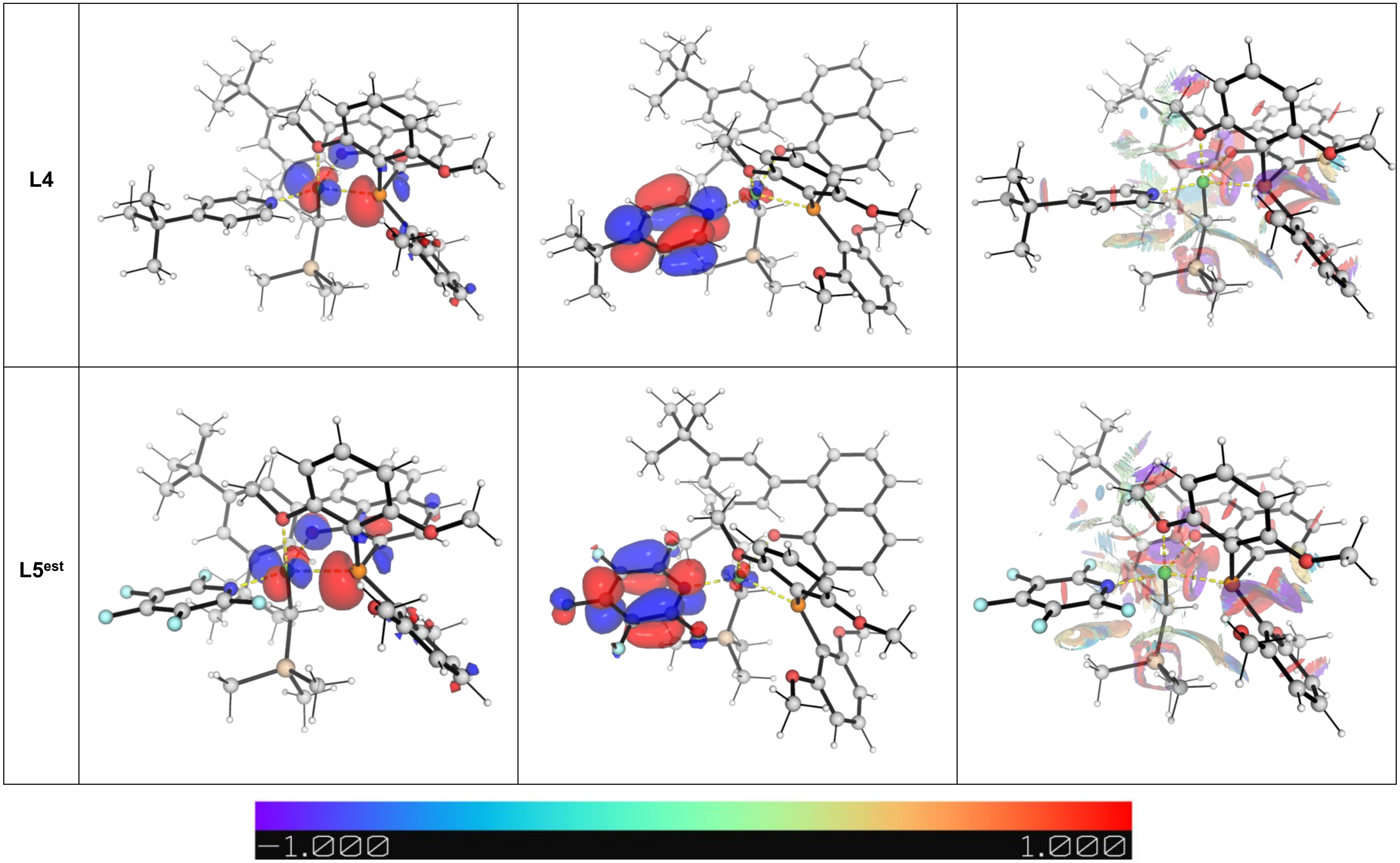

Figure S8. Frontier molecular orbital (FMO) plots including HOMO and LUMO and the non-covalent interaction (NCI) plots for the TSs and estimated TSs.

Table S3. Natural bonding orbitals (NBOs) analysis. NBO charges for key atoms for each system.

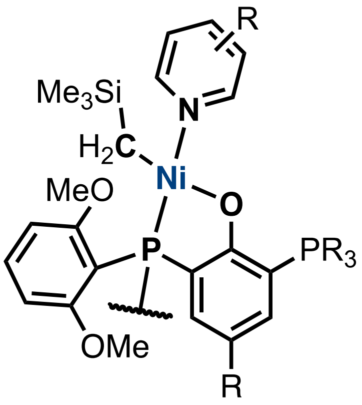

NBO charge (q/e)	Ni	N	C(H_2Me_3)	O(Me)	O(Ph)	P
**TS_5coord_XIII_L1**	0.528	-0.514	-1.133	-0.553	-0.726	0.930
**TS_5coord_XIII_L2**	0.540	-0.528	-1.143	-0.551	-0.726	0.925
**TS_5coord_XIII_L3_est**	0.537	-0.545	-1.139	-0.553	-0.736	0.941
**TS_5coord_XIII_L4**	0.532	-0.518	-1.135	-0.552	-0.725	0.924
**TS_5coord_XIII_L5_est**	0.527	-0.594	-1.131	-0.557	-0.727	0.941
**TS_5coord_XIV_L1**	0.559	-0.514	-1.138	-0.561	-0.709	0.906
**TS_5coord_XIV_L2**	0.608	-0.528	-1.161	-0.566	-0.713	0.881
**TS_5coord_XIV_L3_est**	0.623	-0.552	-1.157	-0.564	-0.714	0.893
**TS_5coord_XIV_L4**	0.560	-0.519	-1.139	-0.560	-0.709	0.905
**TS_5coord_XIV_L5_est**	0.554	-0.595	-1.130	-0.565	-0.710	0.914

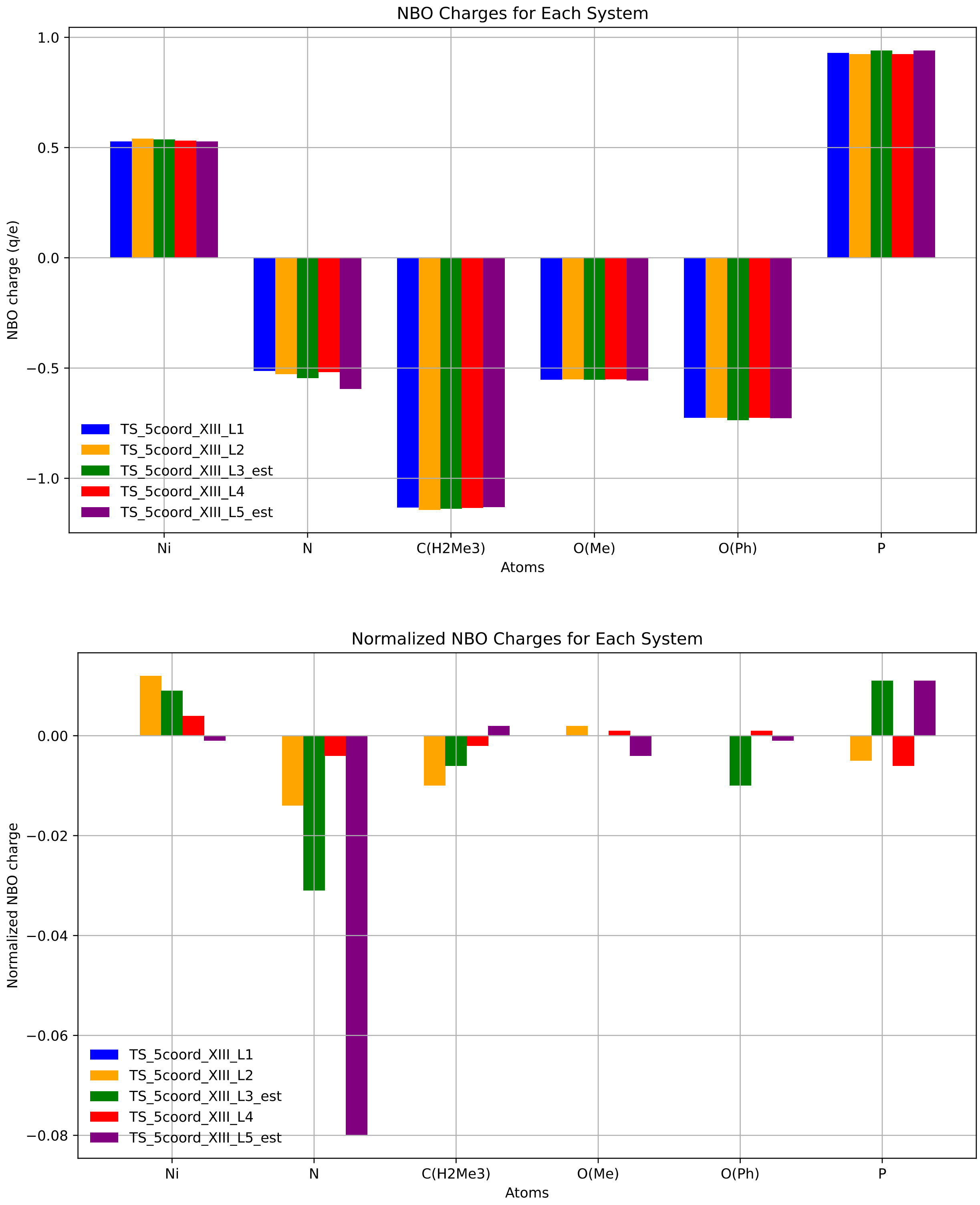

Figure S9. Plot of NBO charges (top) and relative NBO charges (bottom) with respect to pyridine ligand (L1) for catalyst **XIII** system.

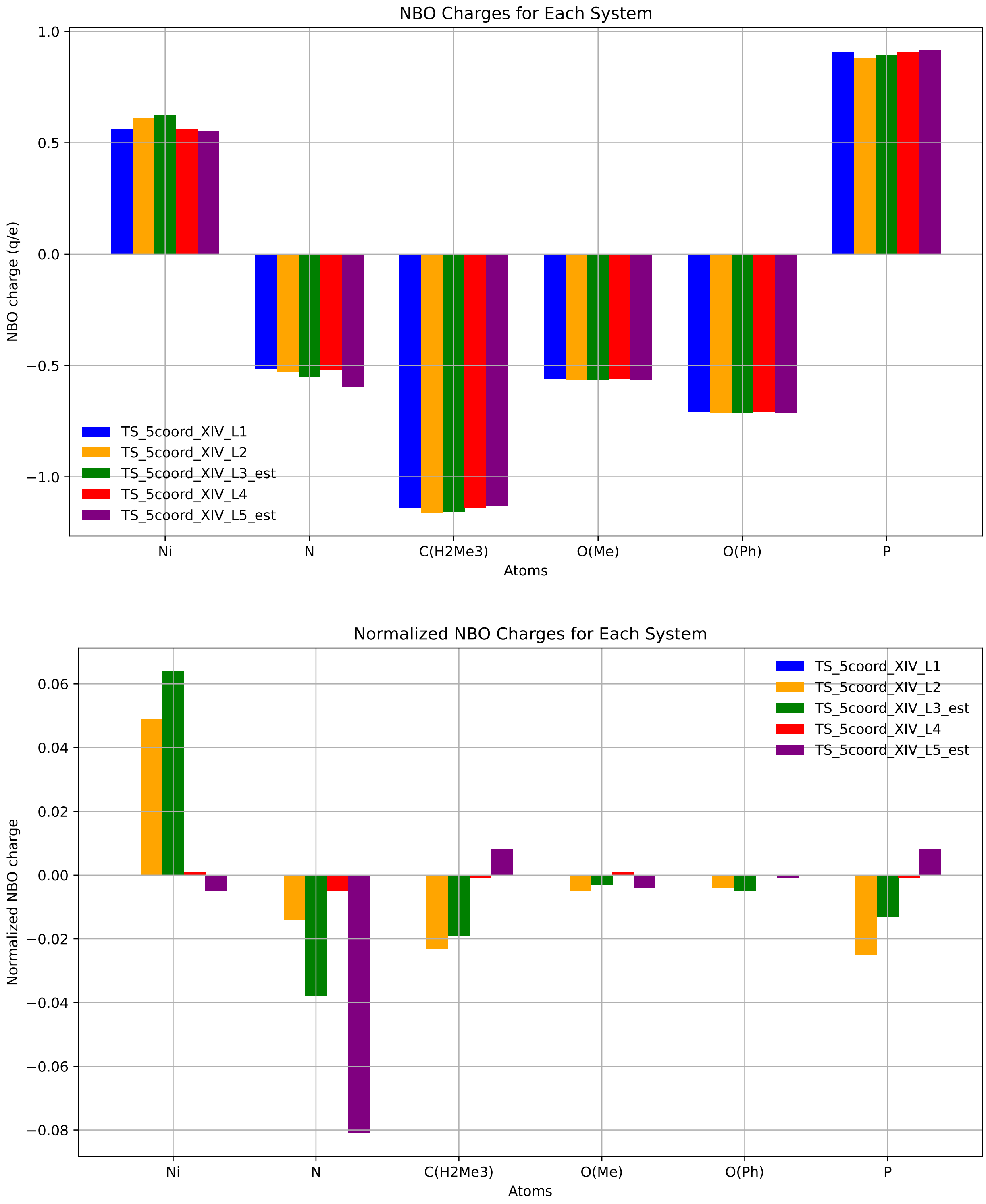

Figure S10. Plot of NBO charges (top) and relative NBO charges (bottom) with respect to pyridine ligand (L1) for catalyst **XIV** system.

Table S4. Natural bonding orbitals (NBOs) analysis. Second-order perturbative stabilization energy, E(2), of selected donor-acceptor (heteroatom to Ni) interactions, in kcal/mol. Donor → acceptor orbitals involved are also shown. CR = core electrons, LP = lone pair, LP* = anti-bonding orbital. [a]Value includes contribution of 13.8 kcal/mol from C–N bonding orbital to LP*.

E2 (kcal/mol)	**N → Ni** N(CR/LP) → Ni LP*	**O(Me) → Ni** O(CR/LP) → Ni LP*	**O(Ph) → Ni** O(CR/LP) → Ni LP*	**P → Ni** P(CR/LP) → Ni LP*
**TS_5coord_XIII_L1**	89.54	64.94	128.03	138.76
**TS_5coord_XIII_L2**	87.85	60.44	129.00	132.11
**TS_5coord_XIII_L3_est**	80.26	66.22	118.79	137.54
**TS_5coord_XIII_L4**	90.86	62.91	131.49	134.63
**TS_5coord_XIII_L5_est**	77.94	63.92	121.59	140.36
**TS_5coord_XIV_L1**	88.58	62.43	138.85	118.15
**TS_5coord_XIV_L2**	86.32	63.90	144.88	92.29
**TS_5coord_XIV_L3_est**	82.68[a]	60.77	128.94	108.87
**TS_5coord_XIV_L4**	89.88	62.15	139.09	116.97
**TS_5coord_XIV_L5_est**	75.74	60.90	137.36	119.73

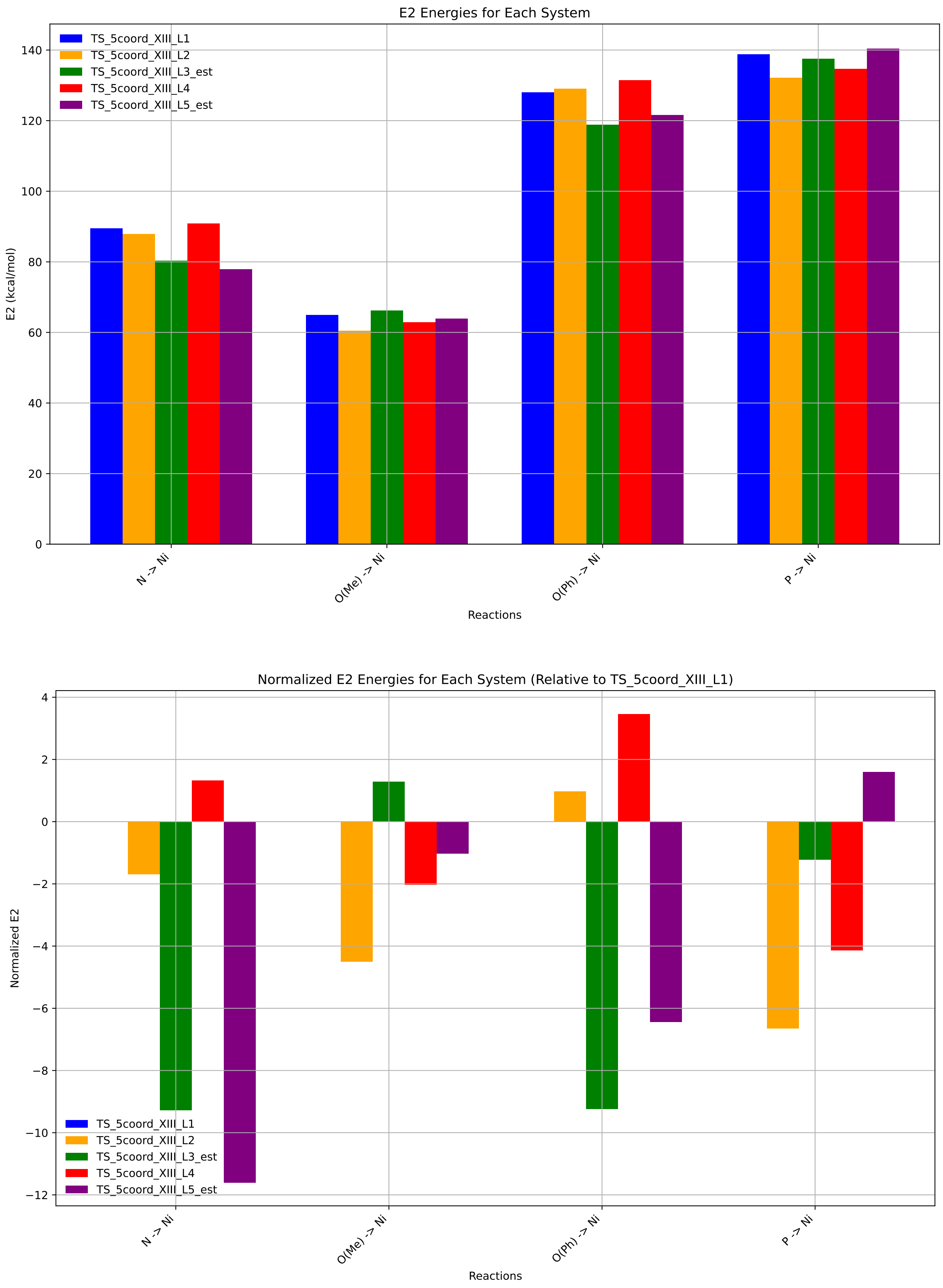

Figure S11. Plot of E2 stabilization energy (top) and relative E2 stabilization energy (bottom) with respect to pyridine ligand (L1) for catalyst **XIII** system.

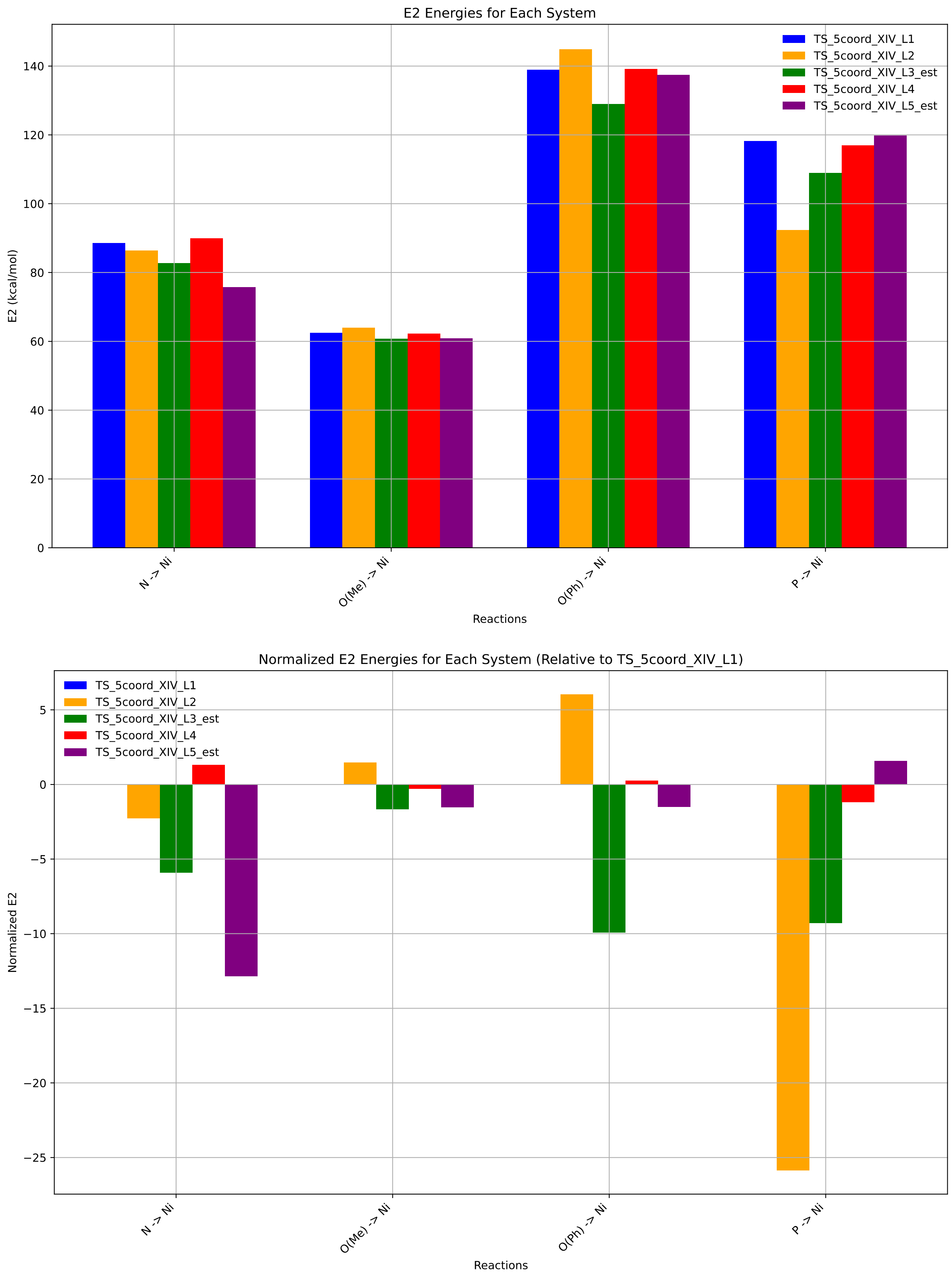

Figure S12. Plot of E2 stabilization energy (top) and relative E2 stabilization energy (bottom) with respect to pyridine ligand (L1) for catalyst **XIV** system.

7. Determination of competing chemical reactivity using simple transition state theory

The Eyring equation

$$k = \frac{k_{\mathrm{B}}T}{h}\mathrm{e}^{-\Delta G^{\ddagger}/RT}$$

gives the rate constant under simple transition state theory (TST) assumptions.

Under kinetic control, as we compare the barrier heights difference between competing transition states, the ratio of the rates between two pathways is given by:

$$\frac{k_A}{k_B} = \frac{e^{-\Delta G_A^{\ddagger}/RT}}{e^{-\Delta G_B^{\ddagger}/RT}} = e^{-\Delta\Delta G^{\ddagger}/RT}$$

where k_X is the rate constant of pathway X (X=A or B); $\Delta G_X^{\ddagger}$ is the activation barrier for pathway X; and $\Delta\Delta G_X^{\ddagger}$ is the difference in the barrier heights; and R is the gas constant, T the temperature. Note that the Eyring Equation pre-exponential factor cancels when comparing the ratio of the rate constants. Thus, using the calculated $\Delta\Delta G_X^{\ddagger}$ value (difference of barrier heights between competing TSs) at the reaction temperature (e.g., 25ºC = 298.15K), we are able to obtain the ratio of competing rates.

8. Absolute contribution to Gibbs energies

Table S5. Absolute values (in Hartrees) for SCF energy, zero-point vibrational energy (ZPE), enthalpy and quasi-harmonic Gibbs free energy (at 50 ºC/323.15 K) for optimized structures are given below. Single point (SP) corrections in SMD chlorobenzene using M06-2X/def2-TZVP level of theory are also included.

Structure	E/au	ZPE/au	H/au	T.S/au	qh-G/au	SP
L1	-247.918857	0.08824	-247.824614	0.032826	-247.857443	-248.190437
L2	-287.178908	0.115015	-287.055799	0.038677	-287.093566	-287.4926071
L3	-326.439448	0.142113	-326.287312	0.042763	-326.329298	-326.795631
L4	-404.935816	0.199027	-404.72448	0.047408	-404.771029	-405.3783343
L5	-743.581282	0.049696	-743.521017	0.04455	-743.565476	-744.4468977
A(XIII)Pyridine-c2	-5189.289395	1.035779	-5188.171824	0.197018	-5188.353102	-5192.79869
A-noL	-4941.317856	0.944147	-4940.297643	0.187362	-4940.469082	-4944.562054
A-noL-c3	-4941.316206	0.944851	-4940.295806	0.184	-4940.46574	-4944.561115

**A(XIII)Pyridine**	-5189.289202	1.035154	-5188.171979	0.197766	-5188.353863	-5192.798603
**A1a**	-5189.256	1.037174	-5188.137348	0.195221	-5188.317992	-5192.765624
**A1b**	-5189.236441	1.035184	-5188.119025	0.20179	-5188.302467	-5192.751538
**A1b-c2**	-5189.237853	1.03536	-5188.120571	0.198891	-5188.302657	-5192.750266
**A(XIII)Pyridine-c3**	-5189.284257	1.036089	-5188.166418	0.196714	-5188.347643	-5192.794419
**A1d-c2**	-5189.243888	1.034564	-5188.12749	0.197767	-5188.309257	-5192.749695
**A1d**	-5189.245263	1.034907	-5188.128507	0.196689	-5188.310061	-5192.751584
**A-et**	-5267.753719	1.08715	-5266.579656	0.209225	-5266.771431	-5271.354506
**B(XIII)Pyridine**	-5189.282916	1.0358	-5188.165403	0.1969	-5188.346632	-5192.792881
**B-noL-c2**	-4941.311226	0.945733	-4940.290402	0.182636	-4940.459158	-4944.555821
**B(XIII)Pyridine-c2**	-5189.282734	1.035474	-5188.165389	0.197968	-5188.347272	-5192.791921
**TS-3coord**	-4941.278469	0.942955	-4940.259822	0.184877	-4940.430272	-4944.527583
**A-noL-c2**	-4941.316206	0.944839	-4940.295813	0.184033	-4940.465765	-4944.56112
**B-noL**	-4941.310354	0.944665	-4940.290057	0.185177	-4940.460407	-4944.554838
**TS_5coord**	-5189.249707	1.035188	-5188.133917	0.192105	-5188.312327	-5192.75566
**TS_5coord-c2**	-5189.248687	1.035727	-5188.132462	0.192582	-5188.310854	-5192.755003
**TS_tet**	-5189.235528	1.033678	-5188.119689	0.200039	-5188.302538	-5192.745177
**TS_tet-c2**	-5189.23483	1.037726	-5188.115745	0.198477	-5188.297268	-5192.745128
**A_XIII_L2**	-5228.550441	1.063053	-5227.403871	0.200367	-5227.58831	-5232.101239
**TS_5coord_L2**	-5228.511037	1.061306	-5227.366886	0.198468	-5227.55021	-5232.05845
**TS_5coord_L2_est**	-5228.510353	1.06104	-5227.368148	0.193059	-5227.547669	-5232.058348
**B_XIII_L2**	-5228.542829	1.063282	-5227.396157	0.199692	-5227.580325	-5232.095132
**A_XIII_L2-c2**	-5228.546245	1.061856	-5227.400272	0.203091	-5227.586706	-5232.098586
**TS_5coord_L2-c2**	-5228.511082	1.061603	-5227.366813	0.196941	-5227.549347	-5232.058177
**TS_5coord_L2-c2_est**	-5228.511612	1.061567	-5227.367268	0.197688	-5227.550093	-5232.058833
**B_XIII_L2-c2**	-5228.541189	1.063217	-5227.394538	0.199684	-5227.578992	-5232.093997

**A_XIII_L3**	-5267.807687	1.08981	-5266.632174	0.204234	-5266.820697	-5271.40139
**TS_5coord_ L3_est**	-5267.769689	1.087918	-5266.597691	0.200276	-5266.783035	-5271.358858
**B_XIII_L3**	-5267.799212	1.089873	-5266.623777	0.205503	-5266.812645	-5271.393496
**A_XIII_L4**	-5346.30802	1.146356	-5345.07338	0.211442	-5345.267013	-5349.988605
**TS_5coord_ L4**	-5346.267523	1.144884	-5345.03507	0.209045	-5345.227261	-5349.944045
**TS_5coord_ L4_est**	-5346.267288	1.144045	-5345.035129	0.212266	-5345.229082	-5349.944166
**B_XIII_L4**	-5346.301301	1.146666	-5345.066684	0.209088	-5345.259091	-5349.982174
**A_XIII_L5**	-5684.946835	0.997639	-5683.86298	0.205947	-5684.052796	-5689.04171
**TS_5coord_ L5_est**	-5684.900487	0.993675	-5683.821879	0.204509	-5684.010001	-5688.9967
**B_XIII_L5**	-5684.933266	0.997797	-5683.84923	0.205912	-5684.039073	-5689.030295
**acrylate**	-423.827572	0.177075	-423.637384	0.049039	-423.685617	-424.3034039
**ethylene**	-78.456815	0.050174	-78.40221	0.025491	-78.427701	-78.54845528
**ts1prime-ac**	-5365.170415	1.126012	-5363.95726	0.206002	-5364.148012	-5368.880371
**int2prime- ac**	-5365.204625	1.126626	-5363.99015	0.210743	-5364.183382	-5368.91406
**ts1-ac**	-5365.159789	1.126414	-5363.946329	0.204982	-5364.136743	-5368.867788
**int2-ac**	-5365.195319	1.12792	-5363.980217	0.206507	-5364.171264	-5368.91406
**ts1-et**	-5019.774153	0.999035	-5018.697015	0.188499	-5018.871365	-5023.101521
**int2-et**	-5019.817207	0.999216	-5018.740402	0.188107	-5018.91399	-5023.143854
**ts1prime-et**	-5019.792277	0.999057	-5018.71502	0.190164	-5018.890033	-5023.120424
**int2prime- et**	-5019.825921	0.999219	-5018.748843	0.189125	-5018.923082	-5023.152336
**int2-ac-py**	-5613.161036	1.217406	-5611.849584	0.222975	-5612.054157	-5617.135497
**int2prime- ac-py**	-5613.163668	1.217455	-5611.852974	0.219067	-5612.055222	-5617.139407
**int2prime- et-py**	-5267.77834	1.090878	-5266.602244	0.207361	-5266.791409	-5271.373589
**int2-et-py**	-5267.769874	1.090707	-5266.59386	0.20564	-5266.782562	-5271.364839
**A_XIV_L1**	-4469.673637	0.971827	-4468.630025	0.17372	-4468.791464	-4472.638968
**TS_tet_XIV**	-4469.608231	0.968904	-4468.566978	0.177022	-4468.730502	-4472.572729
**TS_5coord_ XIV**	-4469.63892	0.970289	-4468.597216	0.17328	-4468.758044	-4472.599126

**INT**	-4469.652191	0.971651	-4468.608577	0.174564	-4468.770367	-4472.614629
**TS2**	-4469.647679	0.971275	-4468.605221	0.173019	-4468.765575	-4472.60984
**B_XIV_L1**	-4469.663848	0.972494	-4468.619926	0.173653	-4468.780789	-4472.62874
**A_XIV_noL**	-4221.708054	0.881447	-4220.761227	0.159922	-4220.910525	-4224.406696
**TS_3coord_ XIV**	-4221.660693	0.878668	-4220.716284	0.162009	-4220.866885	-4224.364393
**B_XIV_noL**	-4221.697519	0.880884	-4220.751063	0.162027	-4220.901211	-4224.395455
**A_XIV_L2**	-4508.929737	0.999418	-4507.852758	0.185366	-4508.025725	-4511.936934
**TS_5coord_ XIV_L2**	-4508.901105	0.997491	-4507.830585	0.17592	-4507.994189	-4511.902433
**TS_5coord_ XIV_L2_est**	-4508.901089	0.996902	-4507.830701	0.179473	-4507.996314	-4511.902583
**B_XIV_L2**	-4508.924112	1.000416	-4507.846476	0.18602	-4508.018908	-4511.930941
**A_XIV_L3**	-4548.190165	1.025699	-4547.088839	0.181188	-4547.257432	-4551.237589
**TS_5coord_ XIV_L3_est**	-4548.151126	1.025309	-4547.050984	0.181068	-4547.213653	-4551.195098
**B_XIV_L3**	-4548.183979	1.026319	-4547.078051	0.190553	-4547.254983	-4551.231994
**A_XIV_L4**	-4626.692368	1.085187	-4625.52988	0.18295	-4625.700647	-4629.829218
**TS_5coord_ XIV_L4**	-4626.656801	1.081225	-4625.497828	0.186303	-4625.670281	-4629.787854
**TS_5coord_ XIV_L4_est**	-4626.656668	1.080403	-4625.498098	0.189073	-4625.672232	-4629.787802
**B_XIV_L4**	-4626.682348	1.082047	-4625.521938	0.188578	-4625.695859	-4629.818582
**A_XIV_L5**	-4965.326991	0.935273	-4964.311447	0.191194	-4964.489943	-4968.875043
**TS_5coord_ XIV_L5_est**	-4965.289716	0.931098	-4964.283177	0.181785	-4964.451765	-4968.838525
**B_XIV_L5**	-4965.315576	0.933776	-4964.301106	0.193693	-4964.480522	-4968.866899

9. Optimised geometries

Geometries of all optimized structures (in .xyz format with their associated energy in Hartrees) are included in a separate folder named *DFT_structures_xyz* with an associated README file. All these data have been deposited with this Supporting Information and uploaded to https://zenodo.org/records/14186325, DOI: 10.5281/zenodo.14186325.

10. References

Full reference for *Guassian 16* software:

Gaussian 16, Revision B.01, M. J. Frisch, G. W. Trucks, H. B. Schlegel, G. E. Scuseria, M. A. Robb, J. R. Cheeseman, G. Scalmani, V. Barone, G. A. Petersson, H. Nakatsuji, X. Li, M. Caricato, A. Marenich, J. Bloino, B. G. Janesko, R. Gomperts, B. Mennucci, H. P. Hratchian, J. V. Ortiz, A. F. Izmaylov, J. L. Sonnenberg, D. Williams-Young, F. Ding, F. Lipparini, F. Egidi, J. Goings, B. Peng, A. Petrone, T. Henderson, D. Ranasinghe, V. G. Zakrzewski, J. Gao, N. Rega, G. Zheng, W. Liang, M. Hada, M. Ehara, K. Toyota, R. Fukuda, J. Hasegawa, M. Ishida, T. Nakajima, Y. Honda, O. Kitao, H. Nakai, T. Vreven, K. Throssell, J. A. Montgomery, Jr., J. E. Peralta, F. Ogliaro, M. Bearpark, J. J. Heyd, E. Brothers, K. N. Kudin, V. N. Staroverov, T. Keith, R. Kobayashi, J. Normand, K. Raghavachari, A. Rendell, J. C. Burant, S. S. Iyengar, J. Tomasi, M. Cossi, J. M. Millam, M. Klene, C. Adamo, R. Cammi, J. W. Ochterski, R. L. Martin, K. Morokuma, O. Farkas, J. B. Foresman, and D. J. Fox. Gaussian, Inc., Wallingford CT, 2016.

References 35–58 are given in the main text.
